# Multiancestry analysis of the HLA locus in Alzheimer’s and Parkinson’s diseases uncovers a shared adaptive immune response mediated by HLA-DRB1*04 subtypes

**DOI:** 10.1073/pnas.2302720120

**Published:** 2023-08-29

**Authors:** Yann Le Guen, Guo Luo, Aditya Ambati, Vincent Damotte, Iris Jansen, Eric Yu, Aude Nicolas, Itziar de Rojas, Thiago Peixoto Leal, Akinori Miyashita, Céline Bellenguez, Michelle Mulan Lian, Kayenat Parveen, Takashi Morizono, Hyeonseul Park, Benjamin Grenier-Boley, Tatsuhiko Naito, Fahri Küçükali, Seth D. Talyansky, Selina Maria Yogeshwar, Vicente Sempere, Wataru Satake, Victoria Alvarez, Beatrice Arosio, Michael E. Belloy, Luisa Benussi, Anne Boland, Barbara Borroni, María J. Bullido, Paolo Caffarra, Jordi Clarimon, Antonio Daniele, Daniel Darling, Stéphanie Debette, Jean-François Deleuze, Martin Dichgans, Carole Dufouil, Emmanuel During, Emrah Düzel, Daniela Galimberti, Guillermo Garcia-Ribas, José María García-Alberca, Pablo García-González, Vilmantas Giedraitis, Oliver Goldhardt, Caroline Graff, Edna Grünblatt, Olivier Hanon, Lucrezia Hausner, Stefanie Heilmann-Heimbach, Henne Holstege, Jakub Hort, Yoo Jin Jung, Deckert Jürgen, Silke Kern, Teemu Kuulasmaa, Kun Ho Lee, Ling Lin, Carlo Masullo, Patrizia Mecocci, Shima Mehrabian, Alexandre de Mendonça, Mercè Boada, Pablo Mir, Susanne Moebus, Fermin Moreno, Benedetta Nacmias, Gael Nicolas, Shumpei Niida, Børge G. Nordestgaard, Goran Papenberg, Janne Papma, Lucilla Parnetti, Florence Pasquier, Pau Pastor, Oliver Peters, Yolande A. L. Pijnenburg, Gerard Piñol-Ripoll, Julius Popp, Laura Molina Porcel, Raquel Puerta, Jordi Pérez-Tur, Innocenzo Rainero, Inez Ramakers, Luis M. Real, Steffi Riedel-Heller, Eloy Rodriguez-Rodriguez, Owen A. Ross, Jose Luís Royo, Dan Rujescu, Nikolaos Scarmeas, Philip Scheltens, Norbert Scherbaum, Anja Schneider, Davide Seripa, Ingmar Skoog, Vincenzo Solfrizzi, Gianfranco Spalletta, Alessio Squassina, John van Swieten, Raquel Sánchez-Valle, Eng-King Tan, Thomas Tegos, Charlotte Teunissen, Jesper Qvist Thomassen, Lucio Tremolizzo, Martin Vyhnalek, Frans Verhey, Margda Waern, Jens Wiltfang, Jing Zhang, EADB, GR@ACE study group, DEGESCO consortium, DemGene, EADI, GERAD, Asian Parkinson’s Disease Genetics consortium, Henrik Zetterberg, Kaj Blennow, Zihuai He, Julie Williams, Philippe Amouyel, Frank Jessen, Patrick G. Kehoe, Ole A. Andreassen, Cornelia Van Duin, Magda Tsolaki, Pascual Sánchez-Juan, Ruth Frikke-Schmidt, Kristel Sleegers, Tatsushi Toda, Anna Zettergren, Martin Ingelsson, Yukinori Okada, Giacomina Rossi, Mikko Hiltunen, Jungsoo Gim, Kouichi Ozaki, Rebecca Sims, Jia Nee Foo, Wiesje van der Flier, Takeshi Ikeuchi, Alfredo Ramirez, Ignacio Mata, Agustín Ruiz, Ziv Gan-Or, Jean-Charles Lambert, Michael D. Greicius, Emmanuel Mignot

**Affiliations:** ^a^Department of Neurology and Neurological Sciences, Stanford University, Stanford 94305, CA; ^b^Institut du Cerveau–Paris Brain Institute–ICM, Paris 75013, France; ^c^Center for Sleep Sciences and Medicine, Stanford University, Palo Alto 94304, CA; ^d^Université de Lille, Inserm, CHU Lille, Institut Pasteur de Lille, U1167-RID-AGE facteurs de risque et déterminants moléculaires des maladies liés au vieillissement, Lille 59000, France; ^e^Department of Neurology, Alzheimer Center Amsterdam, Amsterdam Neuroscience, Vrije Universiteit Amsterdam, Amsterdam UMC, 1081 HV Amsterdam, The Netherlands; ^f^Department of Complex Trait Genetics, Center for Neurogenomics and Cognitive Research, Amsterdam Neuroscience, Vrije University, 1081 HV Amsterdam, The Netherlands; ^g^The Neuro (Montreal Neurological Institute-Hospital), Montreal, Quebec H3A 2B4, Canada; ^h^Department of Human Genetics, McGill University, Montreal, Quebec H3A 0G4, Canada; ^i^Department of Neurology and Neurosurgery, McGill University, Montreal, Quebec H3A 0G4, Canada; ^j^Research Center and Memory clinic Fundació ACE, Institut Català de Neurociències Aplicades, Universitat Internacional de Catalunya, Barcelona 08029, Spain; ^k^Networking Research Center on Neurodegenerative Diseases (CIRNED), Instituto de Salud Carlos III, Madrid 28029, Spain; ^l^Genomic Medicine, Lerner Research Institute, Cleveland Clinic, Cleveland 44196, OH; ^m^Department of Molecular Genetics, Brain Research Institute, Niigata University, Niigata 950-218, Japan; ^n^Lee Kong Chian School of Medicine, Nanyang Technological University Singapore, Singapore 308232, Singapore; ^o^Laboratory of Neurogenetics, Genome Institute of Singapore, A*STAR, Singapore 138672, Singapore; ^p^Division of Neurogenetics and Molecular Psychiatry, Department of Psychiatry and Psychotherapy, Faculty of Medicine and University Hospital Cologne, University of Cologne, Cologne 50937, Germany; ^q^Department of Neurodegenerative diseases and Geriatric Psychiatry, University Hospital Bonn, Medical Faculty, Bonn 53127, Germany; ^r^Medical Genome Center, Research Institute, National Center for Geriatrics and Gerontology, Obu 474-8511, Japan; ^s^Department of Biomedical Science, Chosun University, Gwangju 61452, Korea; ^t^Department of Statistical Genetics, Osaka University Graduate School of Medicine, Suita 565-0871, Japan; ^u^Department of Neurology, Graduate School of Medicine, The University of Tokyo, Tokyo 192-0982, Japan; ^v^Complex Genetics of Alzheimer's Disease Group, VIB Center for Molecular Neurology, VIB, Antwerp 2610, Belgium; ^w^Laboratory of Neurogenetics, Institute Born–Bunge, Antwerp 2610, Belgium; ^x^Department of Biomedical Sciences, University of Antwerp, Antwerp 2000, Belgium; ^y^Department of Neurology, Charité–Universitätsmedizin, Berlin 10117, Germany; ^z^Charité–Universitätsmedizin Berlin, Einstein Center for Neurosciences Berlin, Berlin 10117, Germany; ^aa^Laboratorio de Genética, Hospital Universitario Central de Asturias, Oviedo 33011, Spain; ^bb^Instituto de Investigación Sanitaria del Principado de Asturias, Oviedo 33011, Spain; ^cc^Department of Clinical Sciences and Community Health, University of Milan, Milan 20122, Italy; ^dd^Molecular Markers Laboratory, IRCCS Istituto Centro San Giovanni di Dio Fatebenefratelli, Brescia 25125, Italy; ^ee^Université Paris-Saclay, CEA, Centre National de Recherche en Génomique Humaine, Evry 91057, France; ^ff^Department of Clinical and Experimental Sciences, Centre for Neurodegenerative Disorders, Neurology Unit, University of Brescia, Brescia 25123, Italy; ^gg^Centro de Biología Molecular Severo Ochoa (UAM-CSIC), Universidad Autónoma de Madrid, Madrid 28049, Spain; ^hh^Instituto de Investigacion Sanitaria "Hospital la Paz" (IdIPaz), Madrid 48903, Spain; ^ii^Unit of Neurology, University of Parma and AOU, Parma 43121, Italy; ^jj^Department of Neurology, II B Sant Pau, Hospital de la Santa Creu i Sant Pau, Universitat Autònoma de Barcelona, Barcelona 08193, Spain; ^kk^Department of Neuroscience, Università Cattolica del Sacro Cuore, Rome 00168, Italy; ^ll^Neurology Unit, IRCCS Fondazione Policlinico Universitario A. Gemelli, Rome 00168, Italy; ^mm^University Bordeaux, Inserm, Bordeaux Population Health Research Center, Bordeaux 33000, France; ^nn^Department of Neurology, Bordeaux University Hospital, Bordeaux 33400, France; ^oo^Institute for Stroke and Dementia Research, University Hospital, Ludwig Maximilian University of Munich, 81377, Munich, Germany; ^pp^German Center for Neurodegenerative Diseases, Munich 37075, Germany; ^qq^Munich Cluster for Systems Neurology, Munich 81377, Germany; ^rr^Inserm, Bordeaux Population Health Research Center, UMR 1219, Univ. Bordeaux, ISPED, CIC 1401-EC, Université de Bordeaux, Bordeaux 33405, France; ^ss^CHU de Bordeaux, Pole santé publique, Bordeaux 33400, France; ^tt^German Center for Neurodegenerative Diseases, Magdeburg 39120, Germany; ^uu^Institute of Cognitive Neurology and Dementia Research, Otto-von-Guericke University, Magdeburg 39106, Germany; ^vv^Neurodegenerative Diseases Unit, Fondazione IRCCS Ca’ Granda, Ospedale Policlinico, Milan 20122, Italy; ^ww^Department of Biomedical, Surgical and Dental Sciences, University of Milan, Milan 20122, Italy; ^xx^Hospital Universitario Ramon y Cajal, IRYCIS, Madrid 28034, Spain; ^yy^Alzheimer Research Center and Memory Clinic, Andalusian Institute for Neuroscience, Málaga 29012, Spain; ^zz^Department of Public Health and Caring Sciences, Uppsala University, Uppsala 751 22, Sweden; ^aaa^Geriatrics, Uppsala University, Uppsala 751 22, Sweden; ^bbb^Department of Psychiatry and Psychotherapy, Technical University of Munich, School of Medicine, Klinikum recs der Isar, Munich 80333, Germany; ^ccc^Unit for Hereditary Dementias, Theme Aging, Karolinska University Hospital-Solna, Stockholm 171 64, Swdeen; ^ddd^Department of Child and Adolescent Psychiatry and Psychotherapy, University Hospital of Psychiatry Zurich, University of Zurich, Zurich 8032, Switzerland; ^eee^Neuroscience Center Zurich, University of Zurich and ETH Zurich, Zurich 8057, Switzerland; ^fff^Zurich Center for Integrative Human Physiology, University of Zurich, Zurich 8057, Switzerland; ^ggg^Université de Paris, EA 4468, APHP, Hôpital Broca, Paris 75013, France; ^hhh^Department of Geriatric Psychiatry, Central Institute for Mental Health Mannheim, Faculty Mannheim, University of Heidelberg, Heidelberg 68159, Germany; ^iii^Institute of Human Genetics, University of Bonn, School of Medicine and University Hospital Bonn, Bonn 53127, Germany; ^jjj^Department of Clinical Genetics, VU University Medical Centre, Amsterdam 1081 HV, The Netherlands; ^kkk^Department of Neurology, Memory Clinic, Charles University, 2nd Faculty of Medicine and Motol University Hospital, Prague 150 06, Czech Republic; ^lll^International Clinical Research Center, St. Anne’s University Hospital Brno, Brno 656 91, Czech Republic; ^mmm^Stanford Neurosciences Interdepartmental Program, Stanford University School of Medicine, Stanford 94305, CA; ^nnn^Department of Psychiatry, Psychosomatics and Psychotherapy, Center of Mental Health, University Hospital of Würzburg, Würzburg 97080, Germany; ^ooo^Department of Psychiatry and Neurochemistry, Neuropsychiatric Epidemiology Unit, Institute of Neuroscience and Physiology, the Sahlgrenska Academy, Centre for Ageing and Health (AGECAP) at the University of Gothenburg, Gothenburg 405 30, Sweden; ^ppp^Region Västra Götaland, Sahlgrenska University Hospital, Psychiatry, Cognition and Old Age Psychiatry Clinic, Gothenburg 413 45, Sweden; ^qqq^Institute of Biomedicine, University of Eastern Finland, Joensuu, Kuopio, Eastern Finland 80101, Finland; ^rrr^Department of Biomedical Science, Chosun University, Gwangju 61452, Republic of Korea; ^sss^Department of Integrative Biological Sciences, Chosun University, Gwangju 61452, Republic of Korea; ^ttt^Gwangju Alzheimer's and Related Dementias Cohort Research Center, Chosun University, Gwangju 61452, Republic of Korea; ^uuu^Korea Brain Research Institute, Daegu 41062, Republic of Korea; ^vvv^Neurozen Inc., Seoul 06236, Republic of Korea; ^www^Center for Sleep Sciences and Medicine, Stanford University, Palo Alto 94304, CA; ^xxx^Institute of Neurology, Catholic University of the Sacred Heart, Rome 20123, Italy; ^yyy^Department of Medicine and Surgery, Institute of Gerontology and Geriatrics, University of Perugia, Perugia 06123, Italy; ^zzz^Clinic of Neurology, UH “Alexandrovska”, Medical University–Sofia, Sofia 1431, Bulgaria; ^aaaa^Faculty of Medicine, University of Lisbon, Lisbon 1649-028, Portugal; ^bbbb^Unidad de Trastornos del Movimiento, Servicio de Neurología y Neurofisiología, Instituto de Biomedicina de Sevilla, Hospital Universitario Virgen del Rocío/CSIC/Universidad de Sevilla, Seville 41013, Spain; ^cccc^Institute for Urban Public Health, University Hospital of University Duisburg-Essen, Essen 45147, Germany; ^dddd^Department of Neurology, Hospital Universitario Donostia, San Sebastian 20014, Spain; ^eeee^Neurosciences Area, Instituto Biodonostia, San Sebastian 20014, Spain; ^ffff^Department of Neuroscience, Psychology, Drug Research and Child Health University of Florence, Florence 50121, Italy; ^gggg^IRCCS Fondazione Don Carlo Gnocchi, Florence 20162, Italy; ^hhhh^Department of Genetics and CNR-MAJ, Normandie Univ, UNIROUEN, Inserm U1245 and CHU Rouen, Rouen F-76000, France; ^iiii^Department of Clinical Biochemistry, Copenhagen University Hospital-Herlev Gentofte, Copenhagen 2730, Denmark; ^jjjj^Department of Clinical Medicine, University of Copenhagen, Copenhagen 1172, Denmark; ^kkkk^Department of Neurobiology, Care Sciences and Society, Aging Research Center, Karolinska Institutet and Stockholm University, Stockholm 171 77, Sweden; ^llll^Department of Neurology, Alzheimer Center Erasmus MC, Erasmus University Medical Center, Rotterdam 3000, The Netherlands; ^mmmm^Centre for Memory Disturbances, Lab of Clinical Neurochemistry, Section of Neurology, University of Perugia, Perugia 06123, Italy; ^nnnn^Université de Lille, Inserm 1172, CHU Clinical and Research Memory Research Centre of Distalz, Lille 59000, France; ^oooo^Fundació Docència i Recerca MútuaTerrassa, Terrassa, Barcelona 08221, Spain; ^pppp^Memory Disorders Unit, Department of Neurology, Hospital Universitari Mutua de Terrassa, Terrassa, Barcelona 08221, Spain; ^qqqq^German Center for Neurodegenerative Diseases (DZNE), Berlin 37075, Germany; ^rrrr^Charité–Universitätsmedizin Berlin, Corporate Member of Freie Universität Berlin, Humboldt-Universität zu Berlin, and Berlin Institute of Health, Institute of Psychiatry and Psychotherapy, Berlin 12203, Germany; ^ssss^Unitat Trastorns Cognitius, Hospital Universitari Santa Maria de Lleida, Lleida 25198, Spain; ^tttt^Institut de Recerca Biomedica de Lleida, Lleida 25198, Spain; ^uuuu^Department of Psychiatry, Old Age Psychiatry, Lausanne University Hospital, Lausanne 1005, Switzerland; ^vvvv^Department of Geriatric Psychiatry, University Hospital of Psychiatry Zürich, Zürich 8032, Switzerland; ^wwww^Institute for Regenerative Medicine, University of Zürich, Zürich 8952, Switzerland; ^xxxx^Neurological Tissue Bank–Biobanc- Hospital Clinic-Institut d'Investigacions Biomèdiques August Pi i Sunyer, Barcelona 08036, Spain; ^yyyy^Alzheimer’s disease and other cognitive disorders Unit, Neurology Department, Hospital Clinic, Barcelona 08036, Spain; ^zzzz^Unitat de Genètica Molecular, Institut de Biomedicina de València-Consejo Superior de Investigaciones Científicas Valencia 46010, Spain; ^aaaaa^Unidad Mixta de Neurologia Genètica, Instituto de Investigación Sanitaria La Fe, Valencia 46026, Spain; ^bbbbb^Department of Neuroscience “Rita Levi Montalcini”, University of Torino, Torino 10126, Italy; ^ccccc^Department of Psychiatry and Neuropsychologie, Alzheimer Center Limburg, Maastricht University, Maastricht 6229 GS, The Netherlands; ^ddddd^Unidad Clínica de Enfermedades Infecciosas y Microbiología, Hospital Universitario de Valme, Sevilla 41014, Spain; ^eeeee^Depatamento de Especialidades Quirúrgicas, Bioquímica e Inmunología, Facultad de Medicina, Universidad de Málaga, Málaga 29010, Spain; ^fffff^Institute of Social Medicine, Occupational Health and Public Health, University of Leipzig, Leipzig 04109, Germany; ^ggggg^Neurology Service, Marqués de Valdecilla University Hospital (University of Cantabria and IDIVAL), Santander 39011, Spain; ^hhhhh^Department of Neuroscience, Mayo Clinic-Florida, Jacksonville 32224, FL; ^iiiii^Department of Clinical Genomics, Mayo Clinic-Florida, Jacksonville 32224, FL; ^jjjjj^Depatamento de Especialidades Quirúrgicas, Bioquímica e Inmunología. Facultad de Medicina, Universidad de Málaga, Málaga 29010, Spain; ^kkkkk^Martin-Luther-University Halle-Wittenberg, University Clinic and Outpatient Clinic for Psychiatry, Psychotherapy and Psychosomatics, Halle (Saale) 06120, Germany; ^lllll^Department of Neurology, The Gertrude H. Sergievsky Center, Taub Institute for Research in Alzheimer’s Disease and the Aging Brain, Columbia University, New York 10032, NY; ^mmmmm^1st Department of Neurology, Aiginition Hospital, National and Kapodistrian University of Athens, Medical School, Athens 106 79, Greece; ^nnnnn^Department of Psychiatry and Psychotherapy, Medical Faculty, LVR-Hospital Essen, University of Duisburg-Essen, 45147 Duisberg, Germany; ^ooooo^German Center for Neurodegenerative Diseases (Deutsches Zentrum für Neurodegenerative Erkrankungen), 37075 Göttingen, Germany; ^ppppp^Department for Neurodegenerative Diseases and Geriatric Psychiatry, University Hospital Bonn, Bonn 53127, Germany; ^qqqqq^Department of Hematology and Stem Cell Transplant, Laboratory for Advanced Hematological Diagnostics, Lecce 73100, Italy; ^rrrrr^Neuropsychiatric Epidemiology Unit, Department of Psychiatry and Neurochemistry, Institute of Neuroscience and Physiology, the Sahlgrenska Academy, Centre for Ageing and Health (AGECAP) at the University of Gothenburg, Gothenburg 405 30, Sweden; ^sssss^Interdisciry Department of Medicine, Geriatric Medicine and Memory Unit, University of Bari “A. Moro, Bari 70121, Italy; ^ttttt^Laboratory of Neuropsychiatry, IRCCS Santa Lucia Foundation, Rome 00179, Italy; ^uuuuu^Department of Psychiatry and Behavioral Sciences, Baylor College of Medicine, Houston 77030, TX; ^vvvvv^Department of Biomedical Sciences, University of Cagliari, Cagliari 09124, Italy; ^wwwww^Department of Neurology, ErasmusMC, Rotterdam 3000 CA, Netherlands; ^xxxxx^Alzheimer's disease and other cognitive disorders unit, Service of Neurology, Hospital Clínic of Barcelona, Institut d'Investigacions Biomèdiques August Pi i Sunyer, University of Barcelona, Barcelona 08036, Spain; ^yyyyy^Department of Neurology, National Neuroscience Institute, Singapore General Hospital, Singapore 308433, Singapore; ^zzzzz^Duke-National University of Singapore Medical School, Singapore 169857, Singapore; ^aaaaaa^1st Department of Neurology, Medical school, Aristotle University of Thessaloniki, Thessaloniki 541 24, Greece; ^bbbbbb^Neurochemistry Lab, Department of Clinical Chemistry, Amsterdam Neuroscience, Amsterdam UMC, Vrije Universiteit Amsterdam, Amsterdam 1081 HV, Netherlands; ^cccccc^Department of Clinical Biochemistry, Copenhagen University Hospital–Rigshospitalet, Copenhagen 2100, Denmark; ^dddddd^Neurology, "San Gerardo" hospital, Monza and University of Milano-Bicocca, Monza 20900, Italy; ^eeeeee^Department of Psychiatry and Neuropsychologie, Alzheimer Center Limburg, Maastricht University, Maastricht 6229 GS, Netherlands; ^ffffff^Neuropsychiatric Epidemiology Unit, Department of Psychiatry and Neurochemistry, Institute of Neuroscience and Physiology, the Sahlgrenska Academy, Centre for Ageing and Health (AGECAP) at the University of Gothenburg, Gothenburg 431 41, Sweden; ^gggggg^Region Västra Götaland, Sahlgrenska University Hospital, Psychosis Clinic, Gothenburg 413 45, Sweden; ^hhhhhh^Department of Psychiatry and Psychotherapy, University Medical Center Goettingen, Goettingen 37075, Germany; ^iiiiii^German Center for Neurodegenerative Diseases (Deutsches Zentrum für Neurodegenerative Erkrankungen), Goettingen 37075, Germany; ^jjjjjj^Department of Medical Sciences, Neurosciences and Signaling Group, Institute of Biomedicine, University of Aveiro, Aveiro 3810-193, Portugal; ^kkkkkk^Department of Psychiatry and Neurochemistry, Institute of Neuroscience and Physiology, The Sahlgrenska Academy at the University of Gothenburg, Mölndal 431 41, Sweden; ^llllll^Clinical Neurochemistry Laboratory, Sahlgrenska University Hospital, Mölndal SE-43180, Sweden; ^mmmmmm^Department of Neurodegenerative Disease, UCL Institute of Neurology, London WC1E 6BT, United Kingdom; ^nnnnnn^UK Dementia Research Institute at UCL, London WC1E 6BT, United Kingdom; ^oooooo^Hong Kong Center for Neurodegenerative Diseases, Hong Kong, China; ^pppppp^UKDRI@Cardiff, School of Medicine, Cardiff University, Wales CF14 4YS, United Kingdom; ^qqqqqq^Division of Psychological Medicine and Clinical Neuroscience, School of Medicine, Cardiff University, Cardiff Wales CF14 4XN, United Kingdom; ^rrrrrr^Department of Psychiatry and Psychotherapy, Faculty of Medicine and University Hospital Cologne, University of Cologne, Cologne 50937, Germany; ^ssssss^Cluster of Excellence Cellular Stress Responses in Aging-associated Diseases, University of Cologne, Cologne 50931, Germany; ^tttttt^Translational Health Sciences, Bristol Medical School, University of Bristol, Bristol BS8 1QU, United Kingdom; ^uuuuuu^NORMENT Centre, Division of Mental Health and Addiction, Oslo University Hospital, Oslo 0450, Norway; ^vvvvvv^Institute of Clinical Medicine, University of Oslo, Oslo, Norway; ^wwwwww^Department of Epidemiology, ErasmusMC, Rotterdam 3000 CA, The Netherlands; ^xxxxxx^Nuffield Department of Population Health Oxford University, Oxford OX3 7LF, United Kingdom; ^yyyyyy^Alzheimer’s Centre Reina Sofia-CIEN Foundation, Madrid, Spain; ^zzzzzz^Krembil Brain Institute, University Health Network, Toronto M5G 2C4, Canada; ^aaaaaaa^Department of Medicine and Tanz Centre for Research in Neurodegenerative Diseases, University of Toronto, Toronto M5S 1A8, Canada; ^bbbbbbb^Laboratory of Statistical Immunology, Immunology Frontier Research Center (WPI-IFReC), Osaka University, Suita 565-0871, Japan; ^ccccccc^Integrated Frontier Research for Medical Science Division, Institute for Open and Transdisciplinary Research Initiatives, Osaka University, Suita 565-0871, Japan; ^ddddddd^Center for Infectious Disease Education and Research, Osaka University, Suita 565-0871, Japan; ^eeeeeee^Fondazione IRCCS Istituto Neurologico Carlo Besta, Milan 20133, Italy; ^fffffff^RIKEN Center for Integrative Medical Sciences, Yokohama, Japan; ^ggggggg^Division of Psychological Medicine and Clinical Neuroscience, School of Medicine, Cardiff University, Wales CF14 4YS, United Kingdom; ^hhhhhhh^Department of Psychiatry and Glenn Biggs Institute for Alzheimer’s and Neurodegenerative Diseases, San Antonio 78229, TX

**Keywords:** HLA, Alzheimer’s dementia, Parkinson’s disease, neurodegeneration, autoimmunity

## Abstract

Across multiancestry groups, we analyzed Human Leukocyte Antigen (HLA) associations in over 176,000 individuals with Parkinson’s disease (PD) and Alzheimer’s disease (AD) versus controls. We demonstrate that the two diseases share the same protective association at the HLA locus. HLA-specific fine-mapping showed that hierarchical protective effects of HLA-DRB1*04 subtypes best accounted for the association, strongest with HLA-DRB1*04:04 and HLA-DRB1*04:07, and intermediary with HLA-DRB1*04:01 and HLA-DRB1*04:03. The same signal was associated with decreased neurofibrillary tangles in postmortem brains and was associated with reduced tau levels in cerebrospinal fluid and to a lower extent with increased Aβ42. Protective HLA-DRB1*04 subtypes strongly bound the aggregation-prone tau PHF6 sequence, however only when acetylated at a lysine (K311), a common posttranslational modification central to tau aggregation. An HLA-DRB1*04-mediated adaptive immune response decreases PD and AD risks, potentially by acting against tau, offering the possibility of therapeutic avenues.

## Significance

We report that specific HLA-DRB1*04 alleles are protective against Alzheimer’s dementia (AD), Parkinson’s disease (PD), and other neurodegenerative disorders. Further, we found that these HLA (Human Leukocyte Antigen) subtypes selectively bind a piece of Tau crucial to aggregation but only when it is acetylated (a-PHF6). This a-PHF6 piece is significant as it is a common posttranslational modification of Tau found in Alzheimer’s brains. Only when someone is HLA-DRB1*04:04 or HLA-DRB1*04:01 can PHF6 be presented as a T cell epitope to T cell receptors and mount a memory immune response against this pro-aggregation fragment. This immune response would protect against AD, PD, and neurodegeneration, explaining the HLA association. Vaccination with a-PHF6 in HLA-DRB1*04 individuals could have preventive effects.

Alzheimer’s disease (AD) and Parkinson’s disease (PD) are responsible for considerable morbidity and mortality. Pathophysiology involves the accumulation of tau (neurofibrillary tangles) and Amyloid-β-rich aggregates (amyloid plaques) in AD, and α-synuclein-rich aggregates (Lewy bodies) in PD, although copresence of these aggregates may occur. Consensus is growing that tau may also play a key role in PD (1–3).

Innate immune responses and microglial involvement have long been implicated in neurodegenerative diseases (4–6). More recently, a role for adaptive immunity in PD and AD has also been outlined through genetic (7–10) and immunological studies (11–15). Notably, comparisons of healthy controls and individuals with neurodegenerative diseases have shown that a complex polyclonal T cell response develops against α-synuclein, and tau (11–13). Whether or not these responses are epiphenomenal, contribute to, or protect against neurodegeneration is unknown.

A cornerstone of the adaptive immune response is the highly polymorphic HLA (Human Leukocyte Antigen) locus located on human chromosome 6. *HLA* genes encode a set of proteins that bind peptides derived from foreign or self-antigens, allowing recognition by T cells and subsequent coordination of immune responses. In this context, PD (8) and AD (10) have genome-wide associations in the HLA class II region, within a region containing HLA-DR and DQ, two tightly linked sets of genes. Depending on one’s specific HLA, individuals present distinct repertoires of bound peptides to CD4+ T cells, thus HLA establishes how the immune system sees and reacts to foreign and self-antigens in different individuals.

In PD genome-wide association studies (GWAS), the HLA signal was initially attributed to HLA-DRA, HLA-DRB1*15:01, and *HLA-DRB5* (8), but recent studies indicate it marks HLA-DRB1*04 (16–18). In AD, the HLA signal remains uncharacterized (7, 9, 19) or was assigned to HLA-DRB1*15:01 (7). *SI Appendix*, Table S1 provides a list of previous studies reporting association results at the HLA locus in genetic association studies of AD and PD.

To better understand the involvement of HLA in these diseases, we first gathered genome-wide data available in PD and AD, refined the signal to the HLA subtype level through HIBAG imputation (20), and performed HLA haplotyping to disentangle causal alleles. HLA signals are best dissected across ancestry groups, due to the high variability of HLA alleles across ancestry, as first shown in narcolepsy (21). Therefore, we gathered a large and diverse set of participants including Europeans (10, 17), Asians (22–24), Latin Americans (25), and African Americans (26, 27) to perform fine-mapping of the HLA association in AD and PD. Second, we studied HLA association with pathological observations, finding the strongest protective associations with tau-associated biomarkers. Last, we tested the binding of HLA alleles associated with AD and PD, with epitopes from alpha-synuclein and tau, identifying potential epitopes mediating these effects.

## Results

### Multiancestry Local-GWAS of AD and PD and Colocalization at the HLA Locus.

**Fig. 1.**
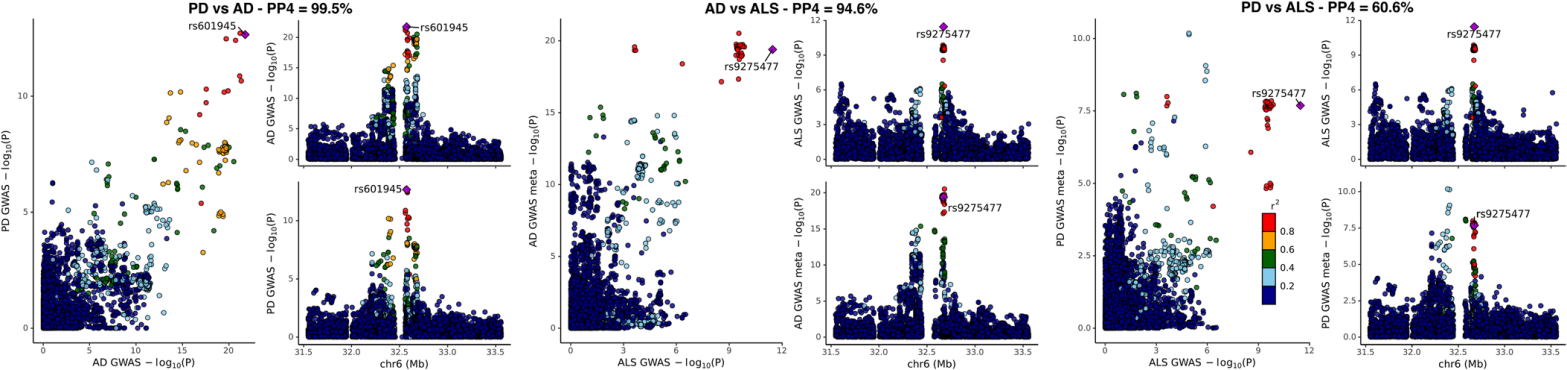
Colocalization of the HLA locus signal in AD, PD, and ALS. PP4: posterior probability of colocalization.

In our AD local-GWAS, 110,927 cases and proxy-cases, and 423,415 controls were included in the meta-analysis (*SI Appendix*, Table S2). Separate analyses were carried out per ancestry and per dataset and meta-analyzed at the HLA locus, with the full GWAS of European and Japanese cohorts now published separately with publicly available summary statistics (10, 24). The most significant association with AD was at rs35472547 (odds ratio [OR] = 0.91; 95% CI, [0.89; 0.93]; *P* = 9.7 × 10^−23^, *SI Appendix*, Fig. S1). In our PD local-GWAS, 41,205 cases and proxy-cases and 474,597 controls were included in the meta-analysis (*SI Appendix*, Table S2). Summary statistics from three published studies (8, 22, 25) were included in this meta-analysis at the HLA locus. The most significant association with PD was at rs504594 (OR = 0.84; 95% CI, [0.80; 0.88]; *P* = 1.83 × 10^−13^, *SI Appendix*, Fig. S1). Colocalization analysis emphasized that the same HLA association signal is shared across AD and PD (posterior probability of colocalization, PP4 = 99.5%, Fig. 1), with rs601945 common to the two GWAS and lead variant in their combination. *SI Appendix*, Figs. S3 and S4 provide locus zoom plots per ancestry of the AD and PD local-GWAS.

### Same Protective HLA Association in Other Neurodegenerative Diseases.

Amyotrophic lateral sclerosis (ALS) GWAS (9) recently reported a genome-wide significant protective association at the HLA locus. Our colocalization analysis shows that this association is shared with AD and PD (PP4 = 94.6%, and PP4 = 60.6%, respectively, Fig. 1 and *SI Appendix*, Fig. S1). The largest Lewy body dementia (LBD) GWAS (28) to date did not contain a genome-wide significant peak at the HLA locus. However, querying rs601945 in this GWAS summary statistics, we noted an association with decreased LBD risk close to nominal significance (OR = 0.91; [0.83; 1.01]; *P* = 0.07) and in the same range as observed in AD and PD, suggesting that a larger sample size may lead to a similarly shared protective association.

### Fine-Mapping of the HLA Association—Allele-, Haplotype-, Amino Acid–Level Analyses—Highlights Association with HLA-DRB1*04 Alleles.

Our HLA-fine-mapping analysis includes a slightly different set of individuals than the local-GWAS. In the AD analysis, 121,411 cases and proxy-cases and 409,096 controls were included (*SI Appendix*, Table S3). In the PD analysis, 55,554 cases and proxy-cases and 1,454,443 controls were included (*SI Appendix*, Table S3). Briefly, all associations were tested under a dominant model as HLA effects are mostly dominant; the presence or absence of an allele allows for recognition of epitopes. All HLA associations from allele-, haplotype-, and amino acid–level analyses are reported in *SI Appendix*, Tables S4 and S5, respectively, for AD and PD, with key findings highlighted in Table 1 and details per cohort in Datasets S1–S5.

**Table 1.** *HLA-DRB1* alleles HLA-DRB1*04:04 and HLA-DRB1*04:01 are associated with a decreased risk of Parkinson’s and ADs

	HLA	PD				AD				PD + AD		
		FreqC	N	OR	*P*-val	FreqC	N	OR	*P*-val	OR	*P*-val	p-het
HLA Alleles	HLA-DRB1*04:01	0.196	1,484,656	0.92[0.89; 0.95]	2.4E-08	0.191	486,478	0.93[0.9; 0.96]	6.4E-07	0.92[0.91; 0.94]	8.9E-14	0.56
	HLA-DRB1*04:02	0.019	1,474,730	0.92[0.85; 0.99]	0.02	0.022	155,846	1.00[0.91; 1.10]	0.99	0.95[0.89; 1.01]	0.07	0.17
	HLA-DRB1*04:03	0.012	980,868	0.89[0.81; 0.97]	0.01	0.072	7,587	1.09[0.91; 1.30]	0.34	0.93[0.85; 1.01]	0.07	0.04
	HLA-DRB1*04:04	0.074	1,475,574	0.84[0.80; 0.88]	1.5E-11	0.073	476,236	0.86[0.82; 0.90]	8.9E-12	0.85[0.82; 0.88]	9.3E-22	0.60
	HLA-DRB1*04:05	0.013	1,507,057	1.00[0.95; 1.05]	0.86	0.026	169,080	0.98[0.91; 1.06]	0.62	0.99[0.95; 1.03]	0.68	0.75
	HLA-DRB1*04:06	0.046	32,327	0.95[0.82; 1.09]	0.46	0.058	7,587	0.95[0.78; 1.15]	0.60	0.95[0.85; 1.06]	0.37	0.99
	HLA-DRB1*04:07	0.021	526,189	0.79[0.69; 0.91]	7.3E-04	0.019	474,840	0.88[0.81; 0.96]	4.5E-03	0.86[0.79; 0.92]	2.7E-05	0.18
	HLA-DRB1*04:10	0.041	4,853	0.91[0.67; 1.25]	0.57	0.031	7,985	1.23[0.94; 1.59]	0.13	1.08[0.89; 1.32]	0.42	0.16
	HLA-DRB1*01:01	0.181	1,485,033	1.05[1.01; 1.09]	7.0E-03	0.186	487,120	1.07[1.04; 1.10]	3.9E-07	1.06[1.04; 1.09]	1.3E-08	0.44
	HLA-DQB1*03:02	0.191	1,501,065	0.91[0.88; 0.93]	2.6E-14	0.190	510,130	0.89[0.86; 0.91]	1.2E-19	0.90[0.88; 0.91]	4.7E-32	0.23
	HLA-DQA1*03:01	0.177	1,507,147	0.89[0.87; 0.91]	2.5E-20	0.186	507,263	0.89[0.87; 0.91]	1.8E-19	0.89[0.88; 0.91]	3.E-37	0.79
	HLA-DRB1*15:01	0.272	1,507,057	1.06[1.03; 1.10]	5.0E-04	0.263	485,383	1.02[0.99; 1.04]	0.13	1.03[1.01; 1.05]	1.1E-03	0.054
HLA Haplotypes	DRB1*04:01~DQA1* 03:01~DQB1*03:02	0.088	1,473,386	0.95[0.92; 0.98]	3.6E-03	0.092	342,115	0.91[0.87; 0.95]	6.5E-06	0.93[0.91; 0.96]	3.2E-07	0.10
	DRB1*04:01~DQA1* 03:03~DQB1*03:01	0.12	1,481,518	0.98[0.97; 0.99]	5.2E-04	0.133	346,157	0.98[0.94; 1.02]	0.36	0.98[0.97; 0.99]	3.4E-04	0.86
	DRB1*04:04~DQA1* 03:01~DQB1*03:02	0.077	1,475,734	0.84[0.80; 0.89]	1.7E-10	0.086	344,760	0.85[0.81; 0.89]	5.4E-11	0.85[0.82; 0.88]	5.7E-20	0.78
	DRB1*04:07~DQA1* 03:01~DQB1*03:02	0.198	1498	0.58[0.44; 0.76]	6.1E-05	0.063	1,865	0.75[0.47; 1.21]	0.23	0.62[0.49; 0.78]	4.6E-05	0.36
	DRB1*04:07~DQA1* 03:03~DQB1*03:01	0.021	524,845	0.87[0.73; 1.04]	0.12	0.017	340,065	0.87[0.79; 0.97]	8.0E-03	0.87[0.80; 0.95]	2.1E-03	0.99
	DRB1*04:05~DQA1* 03:03~DQB1*04:01	0.11	35,369	0.99[0.92; 1.06]	0.76	0.21	4,149	0.97[0.83; 1.13]	0.68	0.99[0.92; 1.05]	0.65	0.81
	DRB1*08:02~DQA1* 03:01~DQB1*03:02	0.026	5,602	1.10[0.76; 1.60]	0.61	0.02	4,149	0.85[0.55; 1.31]	0.46	0.99[0.74; 1.31]	0.93	0.37
	DRB1*01:01~DQA1* 01:01~DQB1*05:01	0.150	1,485,406	1.05[1.01; 1.09]	0.02	0.222	351,276	1.08[1.05; 1.11]	4.8E-07	1.07[1.04; 1.09]	6.8E-08	0.27
Amino acid:	*HLA-DRB1* 13H/33H	0.300	1,507,057	0.89[0.87; 0.91]	9.3E-22	0.315	527,173	0.91[0.89; 0.93]	1.7E-18	0.90[0.89; 0.92]	2.0E-38	0.36

Effect sizes are reported as odds ratio (OR), with 95% CI, and significance (P-value). FreqC: frequency of carriers, N: number of individuals analyzed for a given allele/haplotype/amino acid, p-het: heterogeneity test P-value.

In the HLA-allele-level analysis, the most significant association in AD was observed for HLA-DQB1*03:02 (OR = 0.89[0.86; 0.91]; *P* = 1.2E-19), which was the second most significant allele in PD (OR = 0.91[0.88; 0.93]; *P* = 2.6E-14). In PD, the most significant association was observed for HLA-DQA1*03:01 (OR = 0.89[0.87; 0.91]; *P* = 2.3E-20), which was the second most significant allele in AD (OR = 0.89[0.87; 0.91]; *P* = 1.7E-18). In both AD and PD, HLA-DRB1*15:01 only had a marginal effect (Table 1). It is worth noting that HLA-DQA1*03:01 and DQB1*03:02 are in high linkage disequilibrium and typically found on haplotypes harboring HLA-DRB1*04 subtypes. Because HLA-DRB1*04 alleles are numerous and have more variations, each individually is less common than HLA-DQA1*03:01 and DQB1*03:02; thus, the strength of this association may reflect the sum of HLA-DRB1*04 subtype association.

**Fig. 2.**
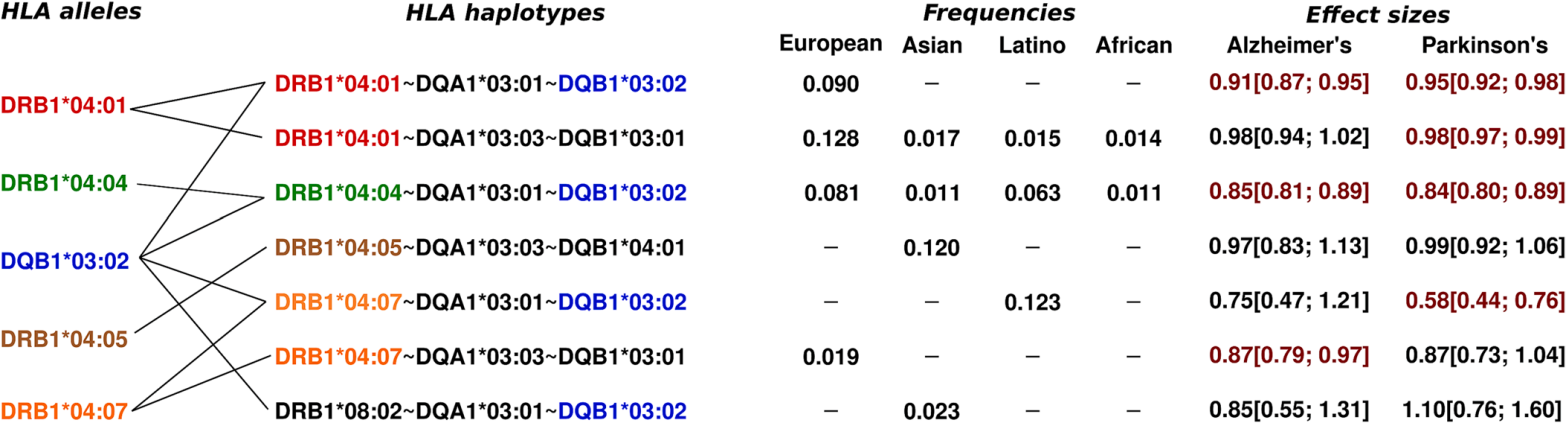
Haplotypes harboring key HLA-DRB1*04 subtypes and/or HLA-DQB1*03:02. Effect sizes highlighted in red were nominally significant (*P* < 0.05).

This is particularly clear in the HLA-haplotype-level analysis, where haplotypes harboring HLA-DRB1*04 subtypes are most significant (Table 1 and *SI Appendix*, Tables S4 and S5). The two significant haplotypes in linkage with DQA1*03:03~DQB1*03:01 are the most interesting since they advocate against the causality of DQA1*03:01~DQB1*03:02 (Fig. 2). Finally, DQA1*03:01~DQB1 *03:02~DRB1*08:02, relatively common in East-Asian populations, does not show any association (Fig. 2), which also argues against DQA1*03:01~DQB1*03:02 causality. Overall, the data suggest hierarchical protective effects of HLA-DRB1*04 subtypes, with *04:04 and *04:07 having the strongest effects on disease protection than other subtypes (Table 1), followed by weaker effects of *04:01, *04:06, and *04:03, and no effect of *04:05.

Last, the HLA-amino acid–level analysis emphasized the pair of *HLA-DRB1* amino acids H13 and H33 as the most significant amino acid changes associated with AD and PD risks (H13 association with AD: OR = 0.91[0.89; 0.93]; *P* = 1.7E-18; and with PD: OR = 0.89[0.87; 0.91]; *P* = 9.3E-22). This pair of amino acid changes is in complete linkage disequilibrium across ancestries and present on all HLA-DRB1*04 subtypes. It is worth emphasizing that the lead variant in the local-GWAS (rs601945) is also in high linkage disequilibrium with these amino acids (D′ close to 1 in Europeans and East-Asians, and R^2^ above 0.9 in Europeans, *SI Appendix*, Fig. S4). Importantly, no significant heterogeneity in effect sizes between AD and PD was observed (Table 1), as formally tested using heterogeneity tests at each allele, haplotype, and amino acid.

### Conditional Analyses Suggest a Shared Association Signal at HLA across AD and PD.

A subset of the participants analyzed with HLA-fine-mapping was also available for conditional analyses using the lead variant (rs601945) and lead amino acid (*HLA-DRB1* H13). In the AD analysis, 120,403 cases and proxy-cases and 408,720 controls were included (*SI Appendix*, Table S6). In the PD analysis, 41,515 cases and proxy-cases and 518,923 controls were included (*SI Appendix*, Table S6). Four conditional analyses were implemented i) local-GWAS conditioned on rs601945, ii) local-GWAS conditioned on *HLA-DRB1* H13, iii) HLA-class II amino acid level conditioned on rs601945, and iv) HLA-class II amino acid level conditioned on H13 (*SI Appendix,* Tables S7 and S8). Overall, the main signal colocalized at the HLA locus across AD and PD disappeared after conditional analyses. Of note, however, whereas no other signals were observed in PD, two independent signals remained genome-wide significant in AD after conditional analysis (*SI Appendix*, Fig. S5). The first significant signal is between *BTLN2* and *HLA-DRA* at lead variant rs3129841 (OR = 0.94[0.92; 0.96]; *P* = 1.0E-08), while the second is between *HLA-DQB1* and *HLA-DQA2* at lead variant rs9275222 (OR = 1.05[1.03; 1.07]; *P* = 6.6E-09). In the HLA-class II amino acid–level conditional analyses, no significant associations were observed in PD (*SI Appendix*, Table S8), while in AD there were several significant associations when conditioning on H13 (*SI Appendix*, Table S7). The two most significant ones were *HLA-DRB1* amino acid changes, N37 (OR = 0.94[0.92; 0.96]; *P* = 6.0E-08) and H32 (OR = 0.94[0.92; 0.96]; *P* = 9.3E-08). However, given the location of the two AD peaks in the conditional analysis (*SI Appendix*, Fig. S5), it seems unlikely that these will be related to these amino acid changes, and other regulatory mechanisms should be explored. In conclusion, the HLA signal in AD is likely more complex than initially described. However, conditional analyses confirmed a shared signal across AD and PD, and this main common signal was the only one pursued in all additional analyses.

### *HLA-DRB1* H13/H33 Is Associated with Decreased Tau Braak Staging.

How could an HLA-DRB1*04 subtype-specific association be involved in AD? To investigate this question, we first used neuropathological information from 7,259 postmortem samples of European ancestry available through the Religious Orders Study and Memory and Aging Project (29) and the National Institute on Aging–AD Center cohorts 1 to 7 (30), looking at the effect of *HLA-DRB1* on tau Braak staging and neuritic plaque density in pathological samples. As shown in Table 2, a significant association of *HLA-DRB1* H13/H33, with decreased neurofibrillary tangles (β = −0.13, 95% CI, [−0.21; −0.05]; *P* = 0.001), but not neuritic plaque density (β = −0.04, 95% CI, [−0.10; 0.02]; *P* = 0.19), was observed, suggesting the involvement of tau. Due to linkage disequilibrium, the same associations were observed for rs601945, and by extension for HLA-DRB1*04 alleles (*SI Appendix*, Tables S9 and S10). Last, in a subset of autopsied individuals with either Lewy Body pathology, AD pathology, or both pathologies, we tested the association of rs601945 with each pathology group versus pathology-free controls. In this comparison, rs601945 was associated with reduced AD only pathology (OR = 0.81[0.69; 0.96], *P* = 0.01) and concordant protective effects in the Lewy Body only pathology group (OR = 0.84[0.69; 1.03], *P* = 0.09) and in the dual pathology group (OR = 0.81[0.59; 1.09], *P* = 0.16), though the last two associations were not significant due to smaller sample sizes in these groups (*SI Appendix*, Table S10).

**Table 2.** *HLA-DRB1* H13/H33 amino acid is associated with reduced tau and neurofibrillary tangles and to a lesser extent with reduced Amyloid-β or neuritic plaques, when testing their association with AD neuropathology and CSF biomarkers

	Phenotype	DRB1 H13			
		N	Freq	β [95% CI]	*P* val
All individuals	Tau Braak staging	7,456	0.293	−0.13[−0.21; −0.05]	1.4E-03
	Neuritic plaques density	5,876	0.292	−0.04[−0.10; 0.02]	0.19
	total-tau in CSF	5,289	0.232	−0.11[−0.17; −0.05]	5.5E-04
	p-tau in CSF	5,269	0.234	−0.08[−0.14; −0.02]	1.0E-02
	Aβ42 in CSF	5,368	0.232	0.08[0.01; 0.14]	0.02
Cases only	Tau Braak staging	5,126	0.283	−0.07[−0.12; −0.01]	0.02
	Neuritic plaques density	4,124	0.289	−0.01[−0.04; 0.02]	0.39
	total-tau in CSF	–	0.228	−0.14[−0.23; −0.06]	8.2E-04
	p-tau in CSF	–	0.228	−0.10[−0.19; −0.01]	0.02
	Aβ42 in CSF	–	0.227	0.09[0.02; 0.16]	1.0E-02
	Age-at-AD-onset	11,900	0.278	0.39[0.03; 0.76]	0.03

p-tau: phosphorylated tau, t-tau: total tau, N: number of individuals, MAF: minor allele frequency, OR: odds ratio, β: parameter estimate, CI: confidence interval. Braak: Tau Braak staging, Neur: Neuritic plaques density.

### *HLA-DRB1* H13/H33 Is Associated with Decreased Tau in CSF and Increased Age-at-Onset.

The analysis of cerebrospinal fluid (CSF) Aβ42 and tau levels in 8,074 subjects of European ancestry independently confirmed this observation (Table 2 and *SI Appendix*, Table S10). In CSF, *HLA-DRB1* H13/H33 was significantly associated with lower levels of phosphorylated- and total-tau (for total tau, β = −0.11, 95% CI, [−0.17; −0.05]; *P* = 0.0006), but the association with increased Aβ42 levels was less significant (β = −0.08, 95% CI, [−0.14; −0.02]; *P* = 0.02). Interestingly, *HLA-DRB1* H13/H33 was also associated with older age-at-onset in AD (β = 0.39, 95% CI, [0.03; 0.76]; *P* = 0.03), as also previously reported in PD (31), further supporting a protective role.

### In Vitro Test of HLA-DRB1*04 Subtypes Binding to Tau and Alpha-Synuclein Peptides Emphasizes the Differential Binding of HLA-DRB1* 04 to Acetylated PHF6 Tau.

**Fig. 3.**
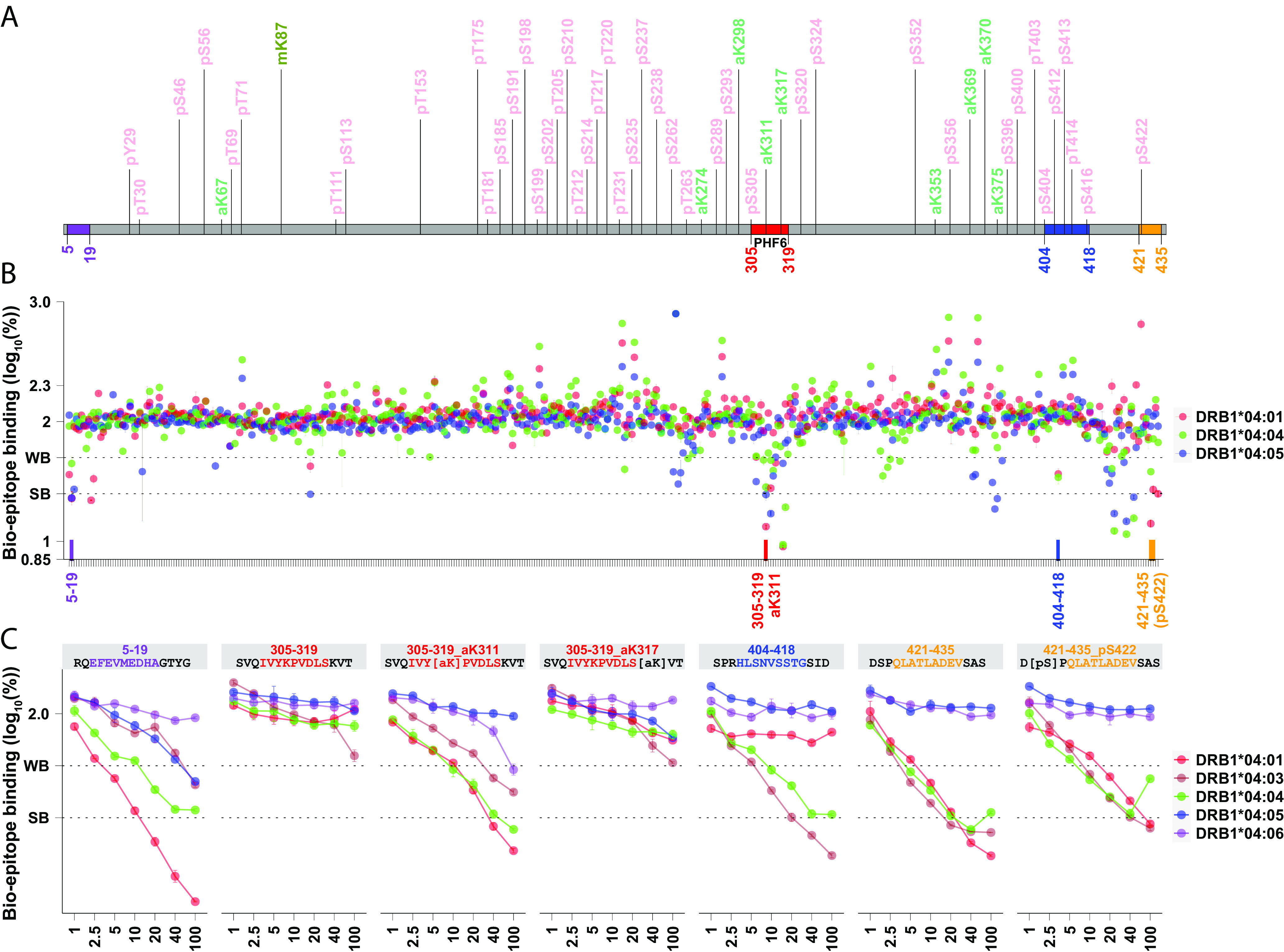
The pro-aggregation PHF6 region of tau binds HLA-DRB1*04 subtypes only when acetylated at K311. Fifteen-mer peptides (800 µM) encompassing the entirety of all tau isoforms (schematized in panel *A*), overlapping across 11 residues, were screened for HLA-DRB1*04:01, HLA-DRB1*04:04 and HLA-DRB1*04:05 binding (*Methods*), with and without common PTMs as reported by Wesseling et al. (32). Four regions (labeled in purple, red, blue, orange, panel *B*) containing strong HLA-DRB1*04 binders (log displacement <1 to 1.4, below 25% of baseline control) were further tested at various concentrations (panel *C*), showing three promising regions where binding was stronger with HLA-DRB1*04:04/ HLA-DRB1*04:01, intermediary with HLA-DRB1*04:03 and absent or weak with HLA-DRB1*04:05 and HLA-DRB1*04:06, a pattern similar to GWAS risk (Table 1). Among these candidate regions, PHF6 ^306^VQIVY(acetylK)PVDLSK^317^ is the only one that strongly binds HLA-DRB1*04:01, HLA-DRB1*04:03, and HLA-DRB1*04:04 and only when posttranslationally modified at K311. This segment is well known to seed tau aggregation, and this process is increased in the presence of acetylK311. Outcompeting a biotinylated epitope (known binder) 75% and 50% is considered as strong binding (SB) and weak binding (WB), respectively. Predicted binding cores are highlighted correspondingly.

Based on these results, we hypothesized that an HLA-DRB1*04-restricted adaptive immune response directed against tau may be protective in AD. To test this hypothesis, we screened the entire tau protein for HLA-RB1*04 subtype-specific binding, using the highly protective HLA-DRB1*04:04 subtype, the moderately protective HLA-DRB1*04:01 subtype, and the neutral HLA-DRB1*04:05 subtype (Fig. 3). Because tau is extensively modified posttranslationally, all most frequent posttranslational modified (PTM) changes (32) were also included, totaling 448 peptides (*SI Appendix*, Table S11). As a control, alpha-synuclein, another extensively PTM protein involved in PD was also tested (*SI Appendix*, Fig. S6) for a total of 62 peptides (*SI Appendix*, Table S12).

Among tested peptides (*SI Appendix*, Tables S11 and S12), only a few peptides strongly bound HLA-DRB1*04 subtypes, with PHF6 in tau (R3/wt; ^306^VQIVY(acetylK)PVDLSKV^318^) standing as the main candidate (Fig. 3). Most interestingly, PHF6 sequences only bind HLA-DRB1*04 when K311 is acetylated (titration repeated 3 times, *SI Appendix*, Fig. S8). Two-way repeated measure ANOVA comparing HLA binding to unacetylated versus acetylated PHF6 was significant for HLA-DRB1*04:01, and HLA-DRB1*04:04 (p_DRB1*04:01_ = 3.58 × 10^−8^, p_DRB1*04:04_ = 1.19 × 10^−4^, *SI Appendix*, Table S13). Additionally, acetylated sequences have significantly less affinity for HLA-DRB1*04:05 versus other subtypes (HLA-DRB1*04:04= HLA-DRB1*04:01> HLA-DRB1* 04:03> HLA-DRB1*04:06> HLA-DRB1*04:05), the same hierarchy observed in the case/control association results, with cores predicted to bind most strongly HLA-DRB1*04:04 (*SI Appendix*, Fig. S7) (33, 34). Additional PTMs in the area, such as acetylated K317, do not alter binding, although the presence of phosphoserine at S305, another frequent PTM, slightly reduces PHF6 binding (Fig. 3). Other peptides were found to bind HLA-DRB1*04 in both tau (Fig. 3) and α-synuclein (*SI Appendix*, Fig. S6), but in no other case was a PTM necessary for epitope binding.

### In Silico Predictions Confirm HLA-DRB1*04 Subtypes as the Unique Common *HLA-DRB1* Alleles Binding to PHF6 Tau.

In-silico predictions confirm that HLA-DRB1*04-associated subtypes are the *only* frequent HLA-DRB1 and DQ subtypes with predicted high affinity for the PHF6 (*SI Appendix*, Fig. S9), likely explaining why HLA-DRB1*04, and no other subtypes, mediate this effect. In-silico predicted binding to the PHF6 motif was also observed with accessory gene HLA-DRB4*01 (*SI Appendix*, Fig. S9). This PHF6/HLA-DRB4*01 strong binding was confirmed in vitro (*SI Appendix*, Fig. S10), but the absence of AD or PD association with HLA-DRB1*07:01 and HLA-DRB1*09:01, whose haplotypes are in linkage with HLA-DRB4*01, ruled out its involvement (*SI Appendix*, Table S14). Last, an absence of predicted binding to PHF6 for DQA1*01:01~DQB1*05:01 or DQA1*03:01~DQB1*03:02 was notable (*SI Appendix*, Fig. S9) and we confirmed this prediction by testing binding in vitro *SI Appendix*, Fig. S10.

## Discussion

Here, we show that HLA HLA-DRB1*04 protects against AD, PD, and probably ALS, three prototypical neurodegenerative diseases and that HLA-DRB1*04 selectively binds the K311-acetylated epitope of the PHF6 sequence of the microtubule-associated protein tau (35), an important region in the mediation of tau aggregates. Presence of HLA-DRB1*04 was also associated with lower CSF tau and fewer neurofibrillary tangles in AD subjects. Although tau aggregation is unlikely to be the sole contributor to neurodegeneration in all these diseases, it may exacerbate pathology in the presence of other aggregates, as has been proposed previously (36, 37).

Tau, like other proteins involved in neurodegeneration, is highly posttranslationally modified (PTM) through e.g. phosphorylation and acetylation, phenomena that likely predispose tau to aggregation (32). In autoimmune diseases, PTMs frequently form part of culprit autoantigens, contributing to reduced self-tolerance as they are not presented in the thymus for negative selection (38). This likely explains the strong polyclonal T cell response observed in controls and AD/PD patients against tau, β-amyloid, and α-synuclein (11, 13, 39). A similarly broad B cell response against tau and α-synuclein is also reported in controls and patients (14, 15). As mentioned above, prior work has outlined strong polyclonal CD4^+^ T cell responses against α-synuclein, β-amyloid, and tau peptides when presented by various HLA subtypes in both cases and controls, thus it is unclear if these responses are effective in limiting disease or are a simple bystander effect (11).

A recent study in humans showed presence of CD4^+^ and CD8^+^ T cells in the CSF of PD and AD patients (40), suggesting CD8^+^ T cell–mediated clearing of amyloid plaques, or a response contributing to neuronal damage. Similarly, Wang et al. recently showed that a microglia-mediated T cell infiltration drives neurodegeneration in a mouse model with tau aggregates; the nefarious adaptive immune response was not observed in mice with amyloid deposition (41). These data suggest that T and B cell responses against tau are common and robust. In our case, we hypothesize that a specific HLA-DRB1*04 restricted response, unlike other adaptive immune responses that may be pathological, targets a particular tau epitope important for the pathological conformation of tau and/or the spreading of aggregates, resulting in a protective effect (*SI Appendix*, Fig. S7). Interestingly, this may be critical to AD, PD, ALS, and other neurodegenerative diseases but not to 4R Tauopathies such as CBD and PSP, given that no large HLA signal has been reported in small sample GWAS of CBD and PSP (42). In line with this hypothesis, in CBD and PSP, acetyl-K311 may not be involved as polyclonal anti-acetyl-K311 antibodies do not recognize the associated tau aggregates (unlike in Pick’s disease and AD, which are 3R or mixed 3/4R pathologies) (43). Further, CryoEM observations suggest that acetyl-K311 is critical to the formation of helical filaments of AD (mix of 3R/4R tau) (44), but not of tau fibrils of CBD (4R tau) (44).

The fact that HLA-DRB1*04 only binds acetylated forms of PHF6 also supports the involvement of this epitope in the protective effect of HLA-DRB1*04 in AD. With K317 located nearby, the K311 PTM of PHF6 is the most differentiating tau PTM found in AD versus control brains. Further, K311 acetylation has been shown by multiple investigators to promote aggregation of PHF6 in vivo (45), in vitro (46) and in silico (43), while K311 carbamylation is inhibitory (47). Crystallography studies have also shown that acetylated PHF6 dominates in the formation of long fibrils as in neurofibrillary tangles of AD (44, 48). PHF6 is also present in all other known forms of tau aggregates identified to date by cryoEM (49–51). It is also needed for all RT-Quick assays of tau (52). Finally, HLA-DRB1*04-associated subtypes are the only frequent HLA-DRB1 and DQ subtypes with predicted high affinity for this epitope (*SI Appendix*, Fig. S9), likely explaining why HLA-DRB1*04, and no other subtypes, mediate this effect. An absence of binding for DQA1*01:01~DQB1*05:01 or DQA1*03:01~DQB1*03:02 is for example notable and reported in *SI Appendix*, Fig. S10. Acetylation at the K311 tau residue may be mediated by SIRT1 and/or HDAC6, current therapeutic targets in AD (45, 53). Similarly, recent evidence suggests that reducing acetylated tau is neuroprotective in brain injury (54).

K311 is not only acetylated, but also methylated, ubiquitinated (32, 55), or succinylated (56), and the epitope is trafficked to the NLRP3 inflammasome of microglial cells (57), where HLA class II presentation of tau fragments by HLA-DRB1*04 to T cells is also likely to occur (58). The involvement of microglial cells in antigen processing and presentation is also suggested by various GWAS association signals observed in AD (4) and PD (5). Although the ubiquitinated K311 epitope is unlikely to bind HLA-DRB1*04 due to steric hindrance at P4, ubiquitination at K317 at P10 could further modulate HLA-DRB1*04 binding and the effect of K311 succinylation or methylation on DRB1*04 binding is unknown. Additional experiments exploring HLA-DRB1*04 subtype binding of PTM segments of PHF6 in various combinations will be needed to further this line of investigation.

Overall, our results indicate that an HLA-DRB1*04-subtype-specific adaptive immune response is protective against both AD and PD. The association with PD is more unexpected, but is in line with recent experiments implicating tau in human PD and in α-synuclein animal models of the disease (1–3, 59). For example, a large inversion that includes the *MAPT* gene encoding tau, is associated with PD (8, 60) and to a lesser extent with AD (10). These and associated polymorphisms are known to modulate tau levels (61), although other effects could be involved as the genetic inversion affects multiple genes and has pleiotropic effects (62, 63). Finally, tau has been involved in multiple other neurodegenerative diseases (37) and in chronic traumatic encephalopathy as well, suggesting it could be a cofactor in multiple brain diseases (64).

**Fig. 4.**
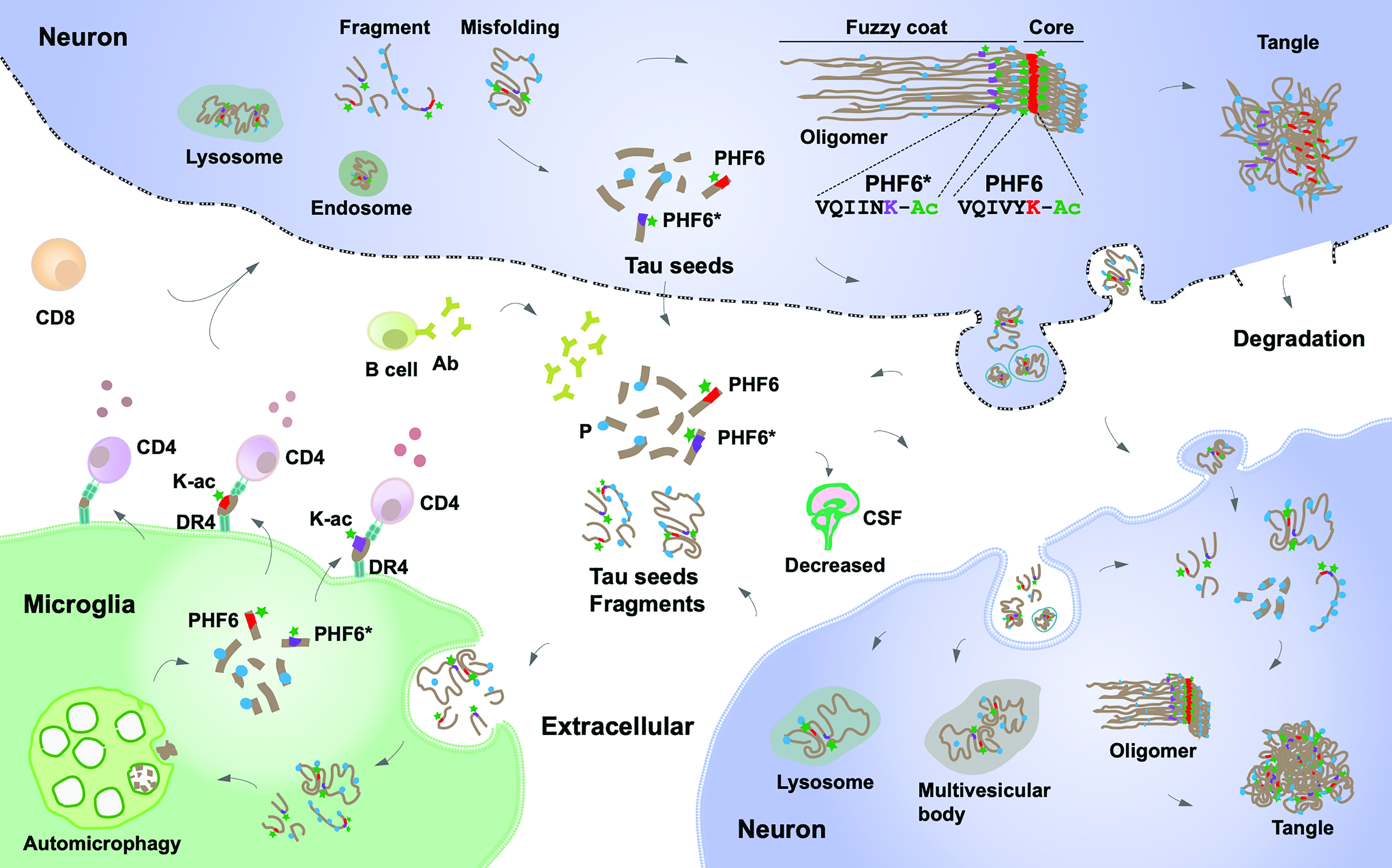
Immune clearance of Acetylated PHF6 tau sequences may reduce neurodegeneration in AD and PD. Pathological tau seeds, soluble tau fragments, or misfolded tau present in the extracellular space are taken up and phagocytosed by activated microglia where it is processed. In addition to autophagy, resulting tau peptide fragments, notably acetylated lysine (K-ac) 311 PHF6 are bound to HLA-DRB1*04:01 or HLA-DRB1*04:04 and the resulting HLA-peptide complexes presented by microglial cells (or other antigen presenting cells) to CD4^+^ T helper cells. CD4^+^ T cells trigger beneficial downstream immune responses perhaps involving CD8^+^ T and antibody producing B cells. These responses limit propagation of misfolded tau and reduce neuropathology, also explaining reduced CSF tau in HLA-DRB1*04 individuals.

Although it is impossible to exclude the involvement of proteins other than tau in the HLA-DRB1*04 observed effect, CD4+ T cell reactivity toward PHF6 fragments containing the acetylated K311 epitope of tau is a strong candidate for mediating most of the effect (Fig. 4). In vitro assays did not yield strong candidates for DRB1*04-mediated effects in alpha-synuclein (for example within the aggregation prone region), although we did identify a strong binding in the C terminus, whose truncation (65) or phosphorylation (66) could additionally modulate alpha-synuclein aggregation. Our results also open the possibility that targeting tau epitopes containing acetylated-K311 through chimeric antigen receptor T cells or antibodies could have therapeutic values. Further, vaccination with acetylated PHF6-like epitopes could reduce disease progression in HLA-DRB1*04 individuals (~20 to 30% of the population across ancestries). It is noteworthy that antibodies, although not targeting acetylated-K311 per se, but adjacent regions within PHF6, were shown to reduce CSF tau and tau pathology in animal models (67) and are being tested as a means of preventing autosomal dominant forms of AD (68).

## Methods

### Samples.

Participants or their caregivers provided written informed consent in the original studies. The current study protocol was granted an exemption by the Stanford University institutional review board because the analyses were carried out on deidentified, off-the-shelf data; therefore, additional informed consent was not required.

The AD samples included in the HLA fine-mapping analysis are part of the following datasets with phenotype, genotype ascertainment, and quality control described elsewhere (7, 10, 23, 24, 26, 69–71): the European AD BioBank (EADB), The Genome Research @ Fundació ACE project (GR@ACE), Genetic and Environmental Risk in AD/Defining Genetic, Polygenic and Environmental Risk for AD Consortium (GERAD), the European AD Initiative (EADI), the Norwegian DemGene (DemGene), the Bonn study (Bonn), the Copenhagen City Heart Study (CCHS), the AD Genetics Consortium (ADGC), the Alzheimer Disease Sequencing Project (ADSP), the UK Biobank, the Gwangju Alzheimer’s and Related Dementias (GARD) study, the Japanese Genetic Study Consortium for AD from Niigata University and National Center for Geriatrics and Gerontology (NCGG). The PD samples included in the HLA fine-mapping analysis are part of the following datasets for which the phenotyping, genotyping, and quality control have been described elsewhere (8, 16, 17, 22, 25, 72): International PD Genomics Consortium (IPDGC) NeuroX dataset, McGill University (McGill), National Institute of Neurological Disorders and Stroke (NINDS) Genome-Wide genotyping in PD, NeuroGenetics Research Consortium (NGRC), Oslo PD Study (Oslo), Parkinson’s Progression Markers Initiative (PPMI), Autopsy-Confirmed Parkinson Disease GWAS Consortium (APDGC), the UK Biobank, East Asians samples from Japan, China, Singapore, Taiwan, and Hong-Kong (EastAsians-PD), 23andMe, and the Latin American Research Consortium on the Genetics of PD (LARGE-PD).

The samples assessed for AD and Lewy-body neuropathology included genetic data from the Rush Religious Orders Study and Memory and Aging Project (70) and from the AD Center cohorts 1 to 7 parts of the ADGC (7), and neuropathological assessment followed procedures described, respectively, in ref. 29 and in the National Alzheimer’s Coordinating Center (NACC) postmortem evaluation protocol (30). The samples with CSF biomarkers included in the analysis for which phenotype, genotype ascertainment, and quality control is described elsewhere (10, 73), are mostly part of the EADB. The remaining of this dataset includes samples originating from the Gothenburg H70 Birth Cohort studies and clinical AD samples from Sweden.

### Genome-Wide Association at the *HLA* Locus and Colocalization between AD and PD.

Given the known signal at HLA in AD GWAS (7, 10) and PD GWAS (8), we aimed at refining the signal at the HLA locus using a multiancestry meta-analysis design. We considered a region, ± 1MB around *HLA-DRB1*, on chromosome 6 from base pair positions (hg38) 31578952 to 33589718. For the PD local-GWAS at the HLA locus, we meta-analyzed the summary statistics from the largest available GWAS to date in European ancestry (8) (distributed without 23andMe), with the Latino-Amerindian GWAS from Kunkle et al. (25) and the Asian GWAS from Kang et al. (22). For the AD local-GWAS at the HLA locus, we meta-analyzed the summary statistics the largest available GWAS to date in European ancestry (10) (which did not include their Stage 2), with the Korean/Japanese GWAS from (24), with in-house local-GWAS at the HLA locus on ADSP and ADGC data carried out by ancestry in European, Latino-Amerindian, African individuals, analyzed with a linear-mixed model as implemented in *GENESIS* (74) (see *Imputation and Statistical Analysis of HLA Alleles, Haplotypes, and Amino Acids* section) adjusted for 6 PCs and sex. All meta-analyses were implemented with a fixed-effect inverse variance weighted design implemented in *METAL* (75). Colocalization between the AD, PD, and ALS, HLA signals in these GWAS was assessed using the Bayesian model implemented in *coloc* (76) using default priors. We report the posterior probability of colocalization (PP4) between two GWAS associations in Fig. 2.

## Imputation and Statistical Analysis of *HLA* Alleles, Haplotypes, and Amino Acids.

Two-field resolution alleles of *HLA-A*, *HLA-B*, *HLA-C* class I genes, and *HLA-DPB1*, *HLA-DQA1*, *HLA-DQB1*, and *HLA-DRB1* class II genes were imputed using R package HIBAG v1.22 (20) for the following dataset: EADB, GR@ACE, GERAD, EADI, DemGene, Bonn, CCHS, UK Biobank, IPDGC, NINDS, NGRC, McGill, Oslo, PPMI, APDGC, LARGE-PD, ADSP, ADGC, GARD, NCGG. When available, training sets specific to ancestry (European, East Asian, Latino, African) and genotyping array were used, either available through HIBAG (20) or trained in-house as previously described (17).

In the allele-level analyses, alleles with an imputation posterior probability lower than 0.5 were considered as undetermined as recommended by HIBAG developers. Each allele was considered as a variant and analyzed under a dominant model; *SI Appendix*, *Supplementary Methods* provide details on the analysis per cohort.

In the haplotype-level analyses, only individuals with nonmissing allele genotypes were included in the haplotype-level analysis. Three-locus HLA class I or class II haplotypes were determined using the haplo.em function from the R haplo.stats package. Only haplotypes with posterior probability >0.5 and a carrier frequency of >1% were included in the analysis. Each haplotype was considered as a variant and analyzed under a dominant model; *SI Appendix*, *Supplementary Methods* provide details on the analysis per cohort.

In the amino acid–level analyses, HIBAG (20) was used to convert P-coded alleles to amino acid sequences for exon 2, 3 of HLA class I genes, and exon 2 of class II genes. Each amino acid was considered as a variant and analyzed under a dominant model; *SI Appendix*, *Supplementary Methods* provide details on the analysis per cohort.

For the East Asians-PD and 23andMe cohorts, the HLA alleles, haplotypes, and amino acids statistics were derived from GWAS summary statistics data using the DISH software (77) as described in ref. 16.

The allele-, haplotype-, amino acid–level analyses were, respectively, meta-analyzed separately between the two neurodegenerative diseases, and across diseases, using a fixed-effect inverse variance weighted design implemented in *METAL* (75).

### Conditional Analyses.

A posteriori conditional analyses were implemented to condition the AD and PD GWAS, on the lead variant common to the two GWAS (rs601945), and separately on the lead amino acid *HLA-DRB1* H13 (in complete linkage disequilibrium with H33). The set of conditional analyses were run the amino acid–level analysis, first conditioning on rs601945 and then conditioning on H13. As for the other analyses, these analyses were run across multiple centers and meta-analyzed with *METAL* (75).

### Tau Peptide Binding.

Competition binding studies were conducted as previously described (78). In brief, tau peptides at different concentrations were incubated with DRB1*04:01, DRB1*04:03, DRB1*04:04, DRB1*04:05, or DRB1*04:06 (from the Emory University NIH core tetramer facility http://tetramer.yerkes.emory.edu/support/faq) for 3 d at 37 °C together with biotinylated GAD or EBV (Bio-GAD, EBV). The reaction was quenched by adding neutralization buffer and then transferred into anti-DR antibody precoated on a 96-well plate. DELFIA^®^ time-resolved fluorescence (TRF) intensity was detected using a Tecan SPARK after adding Europium (Eu)-labeled streptavidin. Nonspecific binding was removed through an extensive wash. Each peptide was duplicated. Competitor tau peptide with Eu TRF intensity that was lower than 25% and 25 to 50% of Bio-GAD or EBV epitope alone was considered a strong binder and a weak binder, respectively. Binding for tau peptides significant for an HLA-DRB1*04 allele (Fig. 2) was repeated three times. Two-way repeated measures ANOVA was used to assess the significance of the differential binding of acetylated vs. unacetylated PHF6 (305-SVQIVY[acetylK]PVDLSKVT-319) across molar ratios.

## Supplementary Material

Appendix 01 (PDF)

Dataset S01 (DOCX)

## Data Availability

All HLA -alleles, -haplotypes, -amino-acid levels associations derived from this study are available per cohort in Datasets S1–S5, as well as the list of tau and alpha-synuclein peptides that were tested for binding.
