# Supplementary material for: Multiancestry analysis of the HLA locus in Alzheimer’s and Parkinson’s diseases uncovers a shared adaptive immune response mediated by HLA-DRB1*04 subtypes
Source: Proc Natl Acad Sci U S A. 2023 Aug 29;120(36):e2302720120. doi: 10.1073/pnas.2302720120 (PMC10483635; doi:10.1073/pnas.2302720120)
Supplement: Supplementary file 1 — Appendix 01 (PDF) [file pnas.2302720120.sapp.pdf]

Supplementary Materials

Le Guen et al. Protective association of *HLA-DRB1**04 subtypes in neurodegenerative diseases implicates acetylated tau PHF6 sequences

# Correspondence should be addressed to:
Prof. Emmanuel Mignot,
Stanford School of Medicine
3165 Porter Drive, #2178, Palo Alto CA 94304, USA
Tel: +1 650-725-6517, Fax: +1 650-725-7341 (fax), Email: mignot@stanford.edu

Dr. Yann Le Guen,
Department of Neurology and Neurological Sciences, Stanford University
290 Jane Stanford Way, E265, CA 94305-5090
Tel: 650 666 2696, Email: yleguen@stanford.edu

Table of Contents

Supplementary Methods……………………………………......

Supplementary Figures……………………………………..……

Supplementary Tables……………………………………..……

Supplementary Acknowledgments………………………….

Consortia author list………………………………...…………..

Supplementary Methods

QUALITY CONTROL AND ANALYSIS PER DATASET

Alzheimer's Disease – ADSP & ADGC datasets

1) Participants and sources of data

Phenotypic information and genotypes were obtained from publicly released genome-wide association study datasets assembled by the Alzheimer's Disease Genetics Consortium (ADGC) and derived from whole-genome sequencing (WGS) data generated by the Alzheimer Disease Sequencing Project (ADSP), with phenotype and genotype ascertainment described elsewhere. The cohorts' queried accession numbers, as well as the sequencing technology or single nucleotide polymorphism (SNP) genotyping platforms are described in **Supplementary Tables 17 and 18**. The microarray datasets are largely part of the ADGC and as such they will be referred thereafter as the ADGC.

2) Quality control procedures

Prior to HLA imputation, ancestry, principal components and relatedness determinations, variants were excluded in each cohort-platform based on genotyping rate (< 95%), MAF < 1%, and Hardy-Weinberg equilibrium in controls ($p < 10^{-6}$) using PLINK v1.9 (1). GnomAD(2) database-derived information was used to filter out SNPs that met one of the following exclusion criteria(3, 4): (i) located in a low complexity region, (ii) located within common structural variants (MAF > 1%), (iii) multiallelic SNPs with MAF > 1% for at least two alternate alleles, (iv) located within a common insertion/deletion, (v) having any flag different than PASS in gnomADv.3, (vi) having potential probe polymorphisms. The latter are defined as SNPs for which the probe may have variable affinity due to the presence of other SNP(s) within 20 bp and with MAF > 1%. Individuals with more than 5% genotype missingness were excluded. Duplicate individuals were identified with KING(5) and their clinical, diagnostic and pathological data (including age-at-onset of cognitive symptoms, age-at-examination for clinical diagnosis, age-at-last exam, age-at-death), as well as sex, race, and *APOE* genotype were cross-referenced across cohorts. Duplicate entries with irreconcilable phenotype or discordant sex were flagged for exclusion.

3) Ancestry determination

For each cohort, we first determined the ancestry of each individual with SNPWeights v2(6) using reference populations from the 1000 Genomes Consortium(7). By applying

an ancestry percentage cut-off > 75%, the samples were stratified into five super populations: South-Asians, East-Asians, Amerindians, Africans, and Europeans, and an Admixed group composed of individuals not passing the 75% cut-off in any single ancestry (**Supplementary Table 18**)(3). The analyses were split into three ancestry groups: Europeans, Africans, and Amerindians-Latinos. The first two groups are composed of individuals passing the 75% threshold in their respective ancestry. The Amerindian-Latinos includes individuals in the Amerindians ancestry group (75% cut-off), and individuals in the Admixed group with at least 15% Amerindians and who identified as Hispanic/Latinos ethnicity. The rationale to include these additional individuals is to compensate the paucity of the Amerindians only group and to have a similar ancestry composition as in the Latin American Research Consortium on the Genetics of Parkinson's Disease (LARGE-PD, see below). Last, enriching for Amerindians ancestry enables us to assess the effect of *HLA-DRB1**04:07 since the HLA haplotype DRB1*04:07~DQA1*03:01~DQB1*03:02 is a common haplotype in this ancestry group.

4) Imputation

Each cohort-genotyping platform was imputed on the TOPMed imputation server per ancestry group to obtain an imputation quality (R^2) per ancestry group. For the local-GWAS at the HLA locus we retained variants with $R^2 > 0.30$, MAF > 1%, and present in 50% of the imputed cohorts.
HLA -alleles and -amino-acids were imputed on platform and ancestry specific reference panels available through HIBAG(8) or trained in-house as previously described(9). In all allele-level analyses, alleles with an imputation posterior probability lower than 0.5 were considered as undetermined as recommended by HIBAG developers, and only allele with carrier frequency above 1% were retained for analysis. For haplotype-level analyses, only individuals with non-missing allele genotypes were included. Three-locus HLA class I or class II haplotypes were determined using the haplo.em function from the R haplo.stats package. Only haplotypes with posterior probability >0.5 and a carrier frequency of >1% were included in the analysis. In the amino-acid-level analyses, HIBAG(8) was used to convert P-coded alleles to amino acid sequences for exons 1-3 of class II genes.

5) Samples retained for analysis

Supplementary Table 19 describes the demographics of individuals retained for analysis. Analyses were implemented into 6 different groups separating WGS data and TOPMed imputed and by ancestry group: ADSP-European, ADSP-African, ADSP-Amerindian-Latino, ADGC-European, ADGC-African, ADGC-Amerindian-Latino.

6) Statistical analyses

In the following paragraph a variable refers indifferently to a variant in the local-GWAS at HLA locus, an HLA-allele, an HLA-haplotype, or any HLA-amino-acids. The AD risk associated with each variable was estimated using a linear mixed model regression on case-control diagnosis. The HLA -allele, -haplotype, and -amino-acids level analyses were run as dominant model (phenotype frequency, collapsing homozygotes for the minor frequency variable with heterozygotes). All statistical analyses were performed in R (v4.0.2) and adjusted for sex, six genetic principal components estimated with the *PC-Air* method(10) implemented in *GENESIS*(11), and covaried by a sparse genetic relationship matrix estimated with the *PC-Relate* method(12) implemented in *GENESIS*. Case-control analyses were not adjusted for age given that controls were older than cases in some subgroups. Correcting for age when cases are younger than controls leads to the model incorrectly inferring the age effect on AD risk, resulting in statistical power loss(3).

Alzheimer's Disease – UK Biobank dataset

1) Participants, quality control and variant imputation

The UK Biobank data includes 488,377 participants which were genotyped on SNP microarrays and imputed at high resolution using two reference panels: (i) the Haplotype Reference Consortium (HRC) for most variants with minor allele frequency > 0.001 and (ii) the UK10K+1000Genomes for variants not in the HRC panel(13). The quality control prior to imputation has been extensively described in Bycroft et al.(13). The proxy-AD phenotype defined in Bellenguez et al.(14) (i.e., cases are individuals who have an ICD10 code linked to AD in their medical record(15) or reported a first degree with Alzheimer's disease, March, 2021 release). We restricted our analysis to 388,051 unrelated individuals after pruning for 3rd degree relatedness using the following criteria to rank order individuals for removal: (i) highest number of relatives, (ii) not a proxy-AD case (iii) and youngest individual.

2) Ancestry determination

Unrelated individuals of the UK Biobank were split into two groups: British and non-British/other ancestries. The British ancestry group corresponds to individuals who self-identified as white British and who clustered on together in the principal ancestry component analysis performed in Bycroft et al. (13)(field ID: 22006). The British ancestry group was composed of 52,426 proxy-AD cases, and 272,624 controls. The non-British/other ancestries group was composed of 7,840 proxy-AD cases and 55,161

controls. This last group was heterogeneous in term of ancestral origin, but most individuals identified as non-British European.

3) HLA Imputation

HLA -alleles and -amino-acids were imputed on platform and ancestry specific reference panels available through HIBAG(8) or trained in-house as previously described(9). In allele-level analyses, alleles with an imputation posterior probability lower than 0.5 were considered as undetermined as recommended by HIBAG developers, and only allele with carrier frequency above 1% were retained for analysis. In the haplotype-level analyses, only individuals with non-missing allele genotypes were included in the haplotype level analysis. Three-locus HLA class I or class II haplotypes were determined using the haplo.em function from the R haplo.stats package. Only haplotypes with posterior probability >0.5 and a carrier frequency of >1% were included in the analysis. In the amino-acid-level analyses, HIBAG(8) was used to convert P-coded alleles to amino acid sequences for exons 1 -3 of class II genes.

4) Statistical analyses

In the following paragraph, a variable refers indifferently to a variant in the local-GWAS at HLA locus, an HLA-allele, an HLA-haplotype, or a specific HLA-amino-acid. HLA -allele, -haplotype, and -amino-acids level analyses were run as dominant model (phenotype frequency, collapsing homozygotes for the minor frequency variable with heterozygotes). Proxy-AD association were tested with plink2 (v2.00a2LM) using the –glm flag covarying for age at last visit, sex, genotyping array, assessment center and the first 20 PCs provided by the UK Biobank.

<u>Alzheimer's Disease – EADB, GR@ACE, GERAD, EADI, DemGene, Bonn, CCHS datasets</u>

Demographics, quality control and GWAS analysis are fully described in Bellengez et al. (14) and demographics are also shown in **Supplementary Table 20**. The HLA analyses were conducted plink2 (v2.00a2LM) using the –glm flag covariates per cohort were described in Bellengez et al. (14).

<u>Alzheimer's Disease – NCGG dataset</u>

The National Center for Geriatrics Gerontology (NCGG) Biobank was established as a geriatric hospital-based Biobank in 2012. The NCGG Biobank is one of the facilities belonging to the National Center Biobank Network. The NCGG Biobank cohort of the study consisted of 2974 patients (female, 64%; mean age, 78.0) with LOAD and 3096 controls (female, 53%; mean age, 71.1) who were recruited from the NCGG Biobank. All subjects were of Japanese origin. Genotyping data were downloaded from the NCGG Biobank database. All subjects were genotyped by using the Affymetrix Japonica Array. Demographics, quality control and GWAS analysis are fully described in Shigemizu et al.(16). The HLA analyses were conducted plink2 (v2.00a2LM) using the –glm flag covariates per cohort were described in Shigemizu et al.(16).

<u>Alzheimer's Disease – GARD dataset</u>

Phenotypic information and genotypes were obtained from the Gwangju Alzheimer's & Related Dementias (GARD) cohort database portal (http://gard.nrcd.re.kr:8080/), with phenotype and genotype ascertainment, as well as ethnical review described elsewhere. Briefly, all cases were LOAD and fulfilled the NINCDS-ADRDA criteria and met the pathological criteria (scanned amyloid beta PET). Genotyping was conducted with the blood species using the Korea Biobank Array, a microarray platform customized for Koreans. Demographics, quality control and GWAS analysis are fully described in Kang et al.(17) and summarized in **Supplementary Table 21**. The HLA analyses were conducted plink2 (v2.00a2LM) using the –glm flag covariates per cohort were described in Kang et al.(17).

<u>Alzheimer's Disease –JGSCAD dataset</u>

Demographics, quality control and GWAS analysis are fully described in Miyashita et al.(18) and summarized in **Supplementary Table 21**. The HLA analyses were conducted

plink2 (v2.00a2LM) using the –glm flag covariates per cohort were described in Miyashita et al.(18).

Alzheimer's Disease Neuropathology – NACC and RUSH datasets

1) Participants and sources of data

Participants were enrolled and followed up at one of Alzheimer's Disease Center (ADC) across the US. Genetic data were obtained from the Rush Religious Orders Study and Memory and Aging Project (ROSMAP)(19) and from the Alzheimer's Disease Center (ADC) cohorts 1 to 7 parts of the ADGC(20) (see **Supplementary Table 17** for data accession number). ROSMAP samples were assessed by the Rush ADC and their neuropathological assessment followed procedures described respectively in Schneider et al.(21). Neuropathological assessment for samples with genotyping from ADGC was obtained from National Alzheimer's Coordinating Center (NACC) and followed postmortem evaluation protocol(22).

2) Quality control procedures, ancestry determination, and imputation

The content of this section is identical to the corresponding sections in "Alzheimer's Disease – ADSP & ADGC datasets" given that these samples were included in the association with AD status.

3) Samples retained for analysis

Supplementary Table 22 describes the demographics of individuals retained for neuropathology analyses: Tau Braak staging, neuritic plaques density. We also defined three categories: AD pathology only, Lewy body (LB) pathology only, and dual pathology (AD and LB) and compared these against controls without AD and LB pathologies. The schematic below describes these categories and follows the classification defined in Tsuang et al.(23).

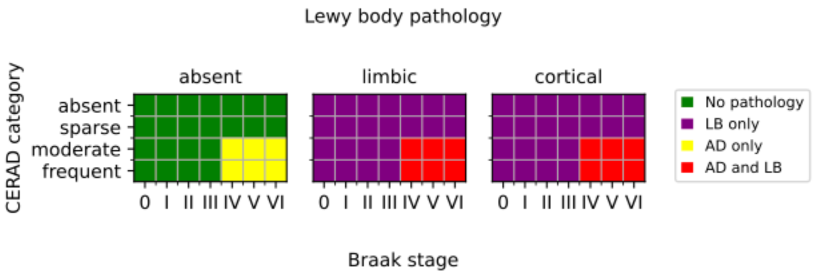

Supplementary Table 23 provides the demographics and number of individuals per category.

4) **Statistical analyses**

The statistical analyses follow the method described in the "Alzheimer's Disease – ADSP & ADGC datasets" corresponding section.

Alzheimer's Disease Cerebrospinal Fluid – EADB and Swedish datasets

1) **Participants and sources of data.**

EADB participants (as described above) for which cerebrospinal fluid (CSF) amyloid beta and/or (phosphorylated) tau measurements were available were included. The Swedish cohorts originate from Gothenburg H70 Birth cohort studies and are clinical AD samples from Sweden all gathered and analyzed in Gothenburg. Genetic data for EADB cohorts has been processed using a consistent approach(14), in which the Illumina Infinium Global Screening Array (GSA, GSAsharedCUSTOM_24+v1.0) was predominantly used in addition to the Axiom 815K Spanish biobank array (Thermo Fisher). The genetic data for the Swedish cohorts were generated with the Illumina Neurochip array.

2) **Quality control procedures and imputation**

Quality control procedures of the EADB datasets are described here in Bellenguez et al(14). For the Swedish datasets, QC and imputation procedures are described elsewhere(24). In short, low-quality variants were excluded based on call rate, minor alle frequency (MAF< 0.01) and Hardy-Weinberg disequilibrium ($P < 1 \times 10^{-6}$). Individuals were removed based on per-sample call rate, sex mismatch, excessive heterozygosity or non-European ancestry. The Sanger imputation service was used to impute post-QC, using the reference panel of Haplotype Reference Consortium data (HRC1.1). The UCSC LiftOver program (https://genome-store.ucsc.edu/) and Plink v2.0 (www.cog-genomics.org/plink/2.0/) were used to lift the GRCh37 genomic positions to GRCh38, the genomic build for all other datasets.

3) **Samples retained for analysis**

Supplementary Table 24 describes the demographics of individuals retained for analysis. The association analyses with HLA haplotypes, alleles and amino acids were only performed for those individuals for which genotype-level data was available (rather

than GWAS summary statistics). For rs601945 association analyses, all cohorts were included.

4) **Statistical analyses**

For HLA-locus, -allele, haplotype, and amino acid association analyses, similar association analysis procedures were performed. For continuous phenotypes Aβ42, tau and pTau, linear regression was performed within each cohort using PLINK v2.0. Association tests were adjusted for gender, age, assay type (if applicable), and ten ancestry principal components. METAL was used for meta-analysis of the per cohort association results, applying the default approach that utilizes p-value and direction of effect, weighted according to sample size.

Association analyses were repeated for subgroups, stratified according to diagnosis status, resulting in a group including only AD subjects, and one including individuals with no or mild cognitive impairment. Covariates were those described for the main analyses above.

<u>Parkinson's Disease – IPDGC, McGill, NINDS, NGRC, Oslo, PPMI, APDGC, UK Biobank datasets</u>

Demographics, phenotyping, quality control, imputation and analysis of the European ancestry cohorts part of local-GWAS at HLA summary statistics have been extensively described in Nalls et al(25). Similarly, the phenotyping, quality control and HLA imputation PD cohorts used in the HLA -alleles, -haplotypes, and -amino-acids level analysis were previously described in Yu et al.(9). Demographics are presented in **Supplementary Table 25**.

<u>Parkinson's Disease – EastAsians-PD and 23andMe datasets</u>

For the EastAsians-PD and 23andMe cohorts, HLA alleles, haplotypes, amino acids statistics were derived from GWAS summary statistics data using the DISH software(26) as described in Naito et al.(27). Demographics, quality control and GWAS analysis were previously described(27–29) and available demographics are reported in **Supplementary Table 26**.

<u>Parkinson's Disease – LARGE-PD dataset</u>

Demographics, quality control and GWAS analysis are fully described in Loesch et al.(30) and demographics are also shown in **Supplementary Table 26**.

References

1. C. C. Chang, *et al.*, Second-generation PLINK: rising to the challenge of larger and richer datasets. *GigaScience* **4**, 7 (2015).

2. K. J. Karczewski, *et al.*, The mutational constraint spectrum quantified from variation in 141,456 humans. *Nature* **581**, 434–443 (2020).

3. Y. Le Guen, *et al.*, A novel age-informed approach for genetic association analysis in Alzheimer's disease. *Alzheimer's Research & Therapy* **13**, 72 (2021).

4. Y. Le Guen, *et al.*, Common X-Chromosome Variants Are Associated with Parkinson Disease Risk. *Annals of Neurology* **90**, 22–34 (2021).

5. A. Manichaikul, *et al.*, Robust relationship inference in genome-wide association studies. *Bioinformatics* **26**, 2867–2873 (2010).

6. C. Y. Chen, *et al.*, Improved ancestry inference using weights from external reference panels. *Bioinformatics* **29**, 1399–1406 (2013).

7. A. Auton, *et al.*, A global reference for human genetic variation. *Nature* **526**, 68–74 (2015).

8. X. Zheng, *et al.*, HIBAG—HLA genotype imputation with attribute bagging. *Pharmacogenomics J* **14**, 192–200 (2014).

9. E. Yu, *et al.*, Fine mapping of the HLA locus in Parkinson's disease in Europeans. *NPJ Parkinsons Dis* **7**, 84 (2021).

10. M. P. Conomos, M. B. Miller, T. A. Thornton, Robust Inference of Population Structure for Ancestry Prediction and Correction of Stratification in the Presence of Relatedness. *Genetic Epidemiology* **39**, 276–293 (2015).

11. S. M. Gogarten, *et al.*, Genetic association testing using the GENESIS R/Bioconductor package. *Bioinformatics* **35**, 5346–5348 (2019).

12. M. P. Conomos, *et al.*, Genetic Diversity and Association Studies in US Hispanic/Latino Populations: Applications in the Hispanic Community Health Study/Study of Latinos. *The American Journal of Human Genetics* **98**, 165–184 (2016).

13. C. Bycroft, *et al.*, The UK Biobank resource with deep phenotyping and genomic data. *Nature* **562**, 203–209 (2018).

14. C. Bellenguez, *et al.*, New insights on the genetic etiology of Alzheimer's and related dementia. *medRxiv*, 2020.10.01.20200659 (2020).

15. I. E. Jansen, *et al.*, Genome-wide meta-analysis identifies new loci and functional pathways influencing Alzheimer's disease risk. *Nature Genetics* **51**, 404–413 (2019).

16. D. Shigemizu, *et al.*, Ethnic and trans-ethnic genome-wide association studies identify new loci influencing Japanese Alzheimer's disease risk. *Transl Psychiatry* **11**, 151 (2021).

17. S. Kang, *et al.*, Potential Novel Genes for Late-Onset Alzheimer's Disease in East-Asian Descent Identified by APOE-Stratified Genome-Wide Association Study. *J Alzheimers Dis* **82**, 1451–1460 (2021).

18. A. Miyashita, *et al.*, SORL1 Is Genetically Associated with Late-Onset Alzheimer's Disease in Japanese, Koreans and Caucasians. *PLOS ONE* **8**, e58618 (2013).

19. D. A. Bennett, *et al.*, Overview and findings from the rush Memory and Aging Project. *Current Alzheimer research* **9**, 646–63 (2012).

20. B. W. Kunkle, *et al.*, Genetic meta-analysis of diagnosed Alzheimer's disease identifies new risk loci and implicates Aβ, tau, immunity and lipid processing. *Nature Genetics* **51**, 414–430 (2019).

21. J. A. Schneider, *et al.*, Cognitive impairment, decline and fluctuations in older community-dwelling subjects with Lewy bodies. *Brain* **135**, 3005–3014 (2012).

22. L. M. Besser, *et al.*, The revised national Alzheimer's coordinating center's neuropathology form-available data and new analyses. *Journal of Neuropathology and Experimental Neurology* **77**, 717–726 (2018).

23. D. Tsuang, *et al.*, APOE ϵ4 increases risk for dementia in pure synucleinopathies. *JAMA Neurology* **70**, 223–228 (2013).

24. J. Najar, *et al.*, Polygenic risk scores for Alzheimer's disease are related to dementia risk in APOE ε4 negatives. *Alzheimer's & Dementia: Diagnosis, Assessment & Disease Monitoring* **13**, e12142 (2021).

25. M. A. Nalls, *et al.*, Identification of novel risk loci, causal insights, and heritable risk for Parkinson's disease: a meta-analysis of genome-wide association studies. *The Lancet Neurology* **18**, 1091–1102 (2019).

26. J. Lim, S.-C. Bae, K. Kim, Understanding HLA associations from SNP summary association statistics. *Sci Rep* **9**, 1337 (2019).

27. T. Naito, *et al.*, Trans-Ethnic Fine-Mapping of the Major Histocompatibility Complex Region Linked to Parkinson's Disease. *Mov Disord* **36**, 1805–1814 (2021).

28. J. N. Foo, *et al.*, Identification of Risk Loci for Parkinson Disease in Asians and Comparison of Risk Between Asians and Europeans: A Genome-Wide Association Study. *JAMA Neurol* **77**, 746–754 (2020).

29. W. Satake, *et al.*, Genome-wide association study identifies common variants at four loci as genetic risk factors for Parkinson's disease. *Nat Genet* **41**, 1303–1307 (2009).

30. D. P. Loesch, *et al.*, Characterizing the Genetic Architecture of Parkinson's Disease in Latinos. *Ann Neurol* **90**, 353–365 (2021).
