# Supplementary material for: Multiancestry analysis of the HLA locus in Alzheimer’s and Parkinson’s diseases uncovers a shared adaptive immune response mediated by HLA-DRB1*04 subtypes
Source: Proc Natl Acad Sci U S A. 2023 Aug 29;120(36):e2302720120. doi: 10.1073/pnas.2302720120 (PMC10483635; doi:10.1073/pnas.2302720120)
Supplement: Supplementary file 2 — Dataset S01 (DOCX) [file pnas.2302720120.sd01.docx]

**Consortia author list**

**EADB**

Céline Bellenguez^1^, Fahri Küçükali^2,3,4^, Iris Jansen^5,6^, Victor Andrade^7,8^, Sonia Moreno-Grau^9,10^, Najaf Amin^11,12^, Benjamin Grenier-Boley^1^, Rafael Campos-Martin^7^, Peter A. Holmans^13^, Anne Boland^14^, Luca Kleineidam^7,8,15^, Vincent Damotte^1^, Sven J. van der Lee^5,16^, Teemu Kuulasmaa^17^, Itziar de Rojas^9,10^, Amber Yaqub^11^, Ivana Prokic^11^, Marcos R, Costa^1,18^, Julien Chapuis^1^, Shahzad Ahmad^11,19^, Vilmantas Giedraitis^20^, Dag Aarsland^21,22^, Pablo Garcia-Gonzalez^9,10^, Carla Abdelnour^9,10^, Emilio Alarcón-Martín^9,23^, Daniel Alcolea^10,24^, Montserrat Alegret^9,10^, Ignacio Alvarez^25,26^, Victoria Álvarez^27,28^, Nicola J. Armstrong^29^, Tsolaki Anthoula^30,31^, Ildebrando Appollonio^32,33^, Marina Arcaro^34^, Silvana Archetti^35^, Alfonso Arias Pastor^36,37^, Beatrice Arosio^38,39^, Lavinia Athanasiu^40^, Henri Bailly^41^, Nerisa Banaj^42^, Miquel Baquero^43^, Ana Belén Pastor^44^, Luisa Benussi^45^, Claudine Berr^46^, Céline Besse^14^, Valentina Bessi^47,48^, Giuliano Binetti^45,49^, Alessandra Bizarro^50^, Rafael Blesa^10,24^, Mercè Boada^9,10^, Barbara Borroni^51^, Silvia Boschi^52^, Paola Bossù^53^, Geir Bråthen^54,55^, Catherine Bresner^13^, Henry Brodaty^29,56^, Keeley J. Brookes^57^, Luis Ignacio Brusco^58,59,60^, Dolores Buiza-Rueda^10,61^, Katharina Bûrger^62,63^, Vanessa Burholt^64,65^, Miguel Calero^10,44,66^, Geneviève Chene^67,68^, Ángel Carracedo^69,70^, Roberta Cecchetti^71^, Laura Cervera-Carles^10,24^, Camille Charbonnier^72^, Caterina Chillotti^73^, Simona Ciccone^39^, Jurgen A.H.R. Claassen^74^, Jordi Clarimon^10,24^, Christopher Clark^75^, Elisa Conti^32^, Anaïs Corma-Gómez^76^, Emanuele Costantini^77^, Carlo Custodero^78^, Delphine Daian^14^, Maria Carolina Dalmasso^7^, Antonio Daniele^77^, Efthimios Dardiotis^79^, Jean-François Dartigues^80^, Peter Paul de Deyn^81^, Stéphanie Debette^80,82^, Jürgen Deckert^83^, Teodoro del Ser^44^, Nicola Denning^84^, Martin Dichgans^62,63,85^, Janine Diehl-Schmid^86^, Mónica Diez-Fairen^25,26^, Paolo Dionigi Rossi^39^, Srdjan Djurovic^40^, Emmanuelle Duron^41^, Emrah Düzel^87,88^, Carole Dufouil^67,68^, Valentina Escott-Price^13,84^, Ana Espinosa^9,10^, Michael Ewers^62,63^, Marta Fernández-Fuertes^76^, Catarina B Ferreira^89^, Evelyn Ferri^39^, Bertrand Fin^14^, Peter Fischer^90^, Tormod Fladby^91^, Klaus Fließbach^8,15^, Juan Fortea^10,24^, Silvia Fostinelli^45^, Nick C. Fox^92^, Emlio Franco-Macías^93^, María J. Bullido^10,94,95^, Ana Frank-García^10,94,96^, Lutz Froelich^97^, Daniela Galimberti^34,88^, Jose Maria García-Alberca^10,98^, Pablo García-González^9^, Sebastian Garcia-Madrona^99^, Guillermo Garcia-Ribas^99^, Roberta Ghidoni^45^, Ina Giegling^100^, Giaccone Giorgio^85^, Oliver Goldhardt^86^, Antonio González-Pérez^101^, Caroline Graff^102,118^, Giulia Grande^103^, Emma Green^104^, Timo Grimmer^86^, Edna Grünblatt^105,106,107^, Tamar Guetta-Baranes^108^, Annakaisa Haapasalo^109^, Georgios Hadjigeorgiou^110^, Harald Hampel^111,112^, Olivier Hanon^41^, John Hardy^113^, Annette M. Hartmann^100^, Lucrezia Hausner^97^, Janet Harwood^13^, Stefanie Heilmann-Heimbach^114^, Seppo Helisalmi^115,116^, Michael T. Heneka^8,16^, Isabel Hernández^9,10^, Martin J. Herrmann^83^, Per Hoffmann^114^, Clive Holmes^117^, Henne Holstege^5,16^, Raquel Huerto Vilas^36,37^, Marc Hulsman^5,16^, Charlotte Johansson^102,118^, Lena Kilander^20^, Anne Kinhult Ståhlbom^102,118^, Miia Kivipelto^119,120,121,122^, Anne Koivisto^115^, Johannes Kornhuber^123^, Mary H. Kosmidis^124^, Carmen Lage^10,125^, Erika J Laukka^103,126^, Alessandra Lauria^50^, Jenni Lehtisalo^115,127^, Ondrej Lerch^128,129^, Alberto Lleó^10,24^, Adolfo Lopez de Munain^10,130^, Malin Löwemark^20^, Lauren Luckcuck^13^, Juan Macías^76^, Catherine A. MacLeod^131^, Wolfgang Maier^8,15^, Francesca Mangialasche^119^, Spallazzi Marco^132^, Marta Marquié^9,10^, Rachel Marshall^13^, Angel Martín Montes^10,94,96^, Carmen Martínez Rodríguez^28^, Carlo Masullo^133^, Simon Mead^134^, Patrizia Mecocci^71^, Miguel Medina^10,44^, Alun Meggy^84^, Shima Mehrabian^135^, Silvia Mendoza^98^, Manuel Menéndez-González^28^, Pablo Mir^10,61^, Susanne Moebus^136^, Merel Mol^137^, Laura Molina-Porcel^138,139^, Laura Montrreal^9^, Laura Morelli^140^, Fermin Moreno^10,130^, Kevin Morgan^141^, Markus M Möthen^114^, Carolina Muchnik^58^, Benedetta Nacmias^47,142^, Tiia Ngandu^127^, Gael Nicolas^72^, Børge G. Nordestgaard^143,144^, Robert Olaso^14^, Adelina Orellana^9,10^, Michela Orsini^77^, Gemma Ortega^9,10^, Alessandro Padovani^51^, Caffarra Paolo^145^, Goran Papenberg^103^, Lucilla Parnetti^87^, Pau Pastor^25,26^, Alba Pérez-Cordón^9^, Jordi Pérez-Tur^10,146,147^, Pierre Pericard^148^, Oliver Peters^149,150^, Yolande A.L. Pijnenburg^5^, Juan A Pineda^76^, Gerard Piñol-Ripoll^36,37^, Claudia Pisanu^151^, Thomas Polak^83^, Julius Popp^152,153,154^, Danielle Posthuma^6^, Josef Priller^150,155^, Raquel Puerta^9^, Olivier Quenez^72^, Inés Quintela^69^, Jesper Qvist Thomassen^156^, Alberto Rábano^10,44^, Innocenzo Rainero^52^, Inez Ramakers^157^, Luis M Real^76,158^, Marcel J.T. Reinders^159^, Steffi Riedel-Heller^160^, Peter Riederer^161^, Natalia Roberto^9^, Eloy Rodriguez-Rodriguez^10,125^, Arvid Rongve^162,163^, Irene Rosas Allende^27,28^, Maitée Rosende-Roca^9,10^, Jose Luis Royo^164^, Elisa Rubino^165^, Dan Rujescu^100^, María Eugenia Sáez^101^, Paraskevi Sakka^166^, Ingvild Saltvedt^55,167^, Ángela Sanabria^9,10^, María Bernal Sánchez-Arjona^93^, Florentino Sanchez-Garcia^168^, Pascual Sánchez Juan^10,125^, Raquel Sánchez-Valle^169^, Sigrid B Sando^54,55^, Michela Scamosci^71^, Nikolaos Scarmeas^170,171^, Elio Scarpini^34,88^, Philip Scheltens^5^, Norbert Scherbaum^172^, Martin Scherer^173^, Matthias Schmid^16,174^, Anja Schneider^8,16^, Jonathan M. Schott^92^, Geir Selbæk^91,175^, Davide Seripa^176^, Alexey A Shadrin^40^, Olivia Skrobot^119^, Hilkka Soininen^115^, Vincenzo Solfrizzi^78^, Alina Solomon^115^, Sandro Sorbi^47,142^, Oscar Sotolongo-Grau^9^, Gianfranco Spalletta^42^, Annika Spottke^16^, Alessio Squassina^177^, Eystein Stordal^178^, Juan Pablo Tartan^9^, Lluís Tárraga^9,10^, Niccolo Tesí^5,16^, Anbupalam Thalamuthu^29^, Tegos Thomas^30,31^, Latchezar Traykov^135^, Lucio Tremolizzo^32,33^, Anne Tybjærg-Hansen^144,156^, Andre Uitterlinden^179^, Abbe Ullgren^102^, Ingun Ulstein^175^, Sergi Valero^9,10^, Aad van der Lugt^180^, Jasper Van Dongen^2,3,4^, Jeroen van Rooij^137^, John van Swieten^137^, Rik Vandenberghe^181,182^, Frans Verhey^157^, Jean-Sébastien Vidal^41^, Jonathan Vogelgsang^183,184^, Martin Vyhnalek^128,129^, Michael Wagner^8,16^, David Wallon^185^, Leonie Weinhold^174^, Jens Wiltfang^183,186,187^, Gill Windle^131^, Bob Woods^131^, Mary Yannakoulia^188^, Miren Zulaica^10,189^, Jan Laczo^128,129^, Vaclav Matoska^190^, Maria Serpente^88^, Francesca Assogna^42^, Fabrizio Piras^42^, Federica Piras^42^, Valentina Ciullo^42^, Jacob Shofany^42^, Carlo Ferrarese^32,33^, Simona Andreoni^32^, Gessica Sala^32^, Chiara Paola Zoia^32^, Maria Del Zompo^177^, Alberto Benussi^51^, Patrizia Bastiani^191^, Mari Takalo*^192^, Teemu Natunen*^192^, Tiina Laatikainen^120,127^, Jaakko Tuomilehto^120,127^, Riitta Antikainen^193,194^, Timo Strandberg^193,195^, Jaana Lindström^127^, Markku Peltonen^127^, Richard Abraham^196^, Ammar Al-Chalabi^197^, Nicholas J. Bass^198^, Carol Brayne^199^, Kristelle S. Brown^200^, John Collinge^201^, David Craig^202^, Pangiotis Deloukas^203^, Nick Fox^204^, Amy Gerrish^204^, Michael Gill^205^, Rhian Gwilliam^203^, John Hardy^206^, Denise Harold^207^, Paul Hollingworth^196^, Jarret A, Johnston^208^, Lesley Jones^196^, Brian Lawlor^205^, Gill Livingston^198^, Simon Lovestone^209^, Michelle Lupton^210,211^, Aoibhinn Lynch^205^, David Mann^212^, Bernadette McGuinness^208^, Andrew McQuillin^198^, Michael C. O’Donovan^196^, Michael J. Owen^196^, Peter Passmore^208^, John F, Powell^210,211^, Petra Proitsi^210,211^, Martin Rossor^204^, Christopher E. Shaw^197^, A. David Smith^213^, Hugh Gurling^214^, Stephen Todd^215^, Catherine Mummery^216^, Nathalie Ryan^216^, Giordano Lacidogna^77^, Ad Adarmes-Gómez^10,61^, Ana Mauleón^9^, Ana Pancho^9^, Anna Gailhajenet^9^, Asunción Lafuente^9^, D Macias-García^10,61^, Elvira Martín^9^, Esther Pelejà^9^, F Carrillo^10,61^, Isabel Sastre Merlín^10,95^, L Garrote-Espina^10,61^, Liliana Vargas^9^, M Carrion-Claro^10,61^, M Marín^93^, Ma Labrador^10,61^, Mar Buendia^9^, María Dolores Alonso^217^, Marina Guitart^9^, Mariona Moreno^9^, Marta Ibarria^9^, Mt Periñán^10,61^, Nuria Aguilera^9^, P Gómez-Garre^10,61^, Pilar Cañabate^9^, R Escuela^10,61^, R Pineda-Sánchez^10,61^, R Vigo-Ortega^10,61^, S Jesús^10,61^, Silvia Preckler^9^, Silvia Rodrigo-Herrero^93^, Susana Diego^9^, Alessandro Vacca^52^, Fausto Roveta^52^, Nicola Salvadori^87^, Elena Chipi^87^, Henning Boecker^15,218^, Christoph Laske^219,220^, Robert Perneczky^65,221^, Costas Anastasiou^188^, Daniel Janowitz^62^, Rainer Malik^62^, Anna Anastasiou^30^, Kayenat Parveen^7^, Carmen Lage^222^, Sara López-García^222^, Anna Antonell^169^, Kalina Yonkova Mihova^223^, Diyana Belezhanska^135^, Heike Weber^224^, Silvia Kochen^225^, Patricia Solis^225^, Nancy Medel^225^, Julieta Lisso^225^, Zulma Sevillano^225^, Daniel G Politis^225,226^, Valeria Cores^225,226^, Carolina Cuesta^225,226^, Cecilia Ortiz^227^, Juan Ignacio Bacha^227^, Mario Rios^228^, Aldo Saenz^228^, Mariana Sanchez Abalos^229^, Eduardo Kohler^230^, Dana Lis Palacio^231^, Ignacio Etchepareborda^231^, Matias Kohler^231^, Gisela Novack^232^, Federico Ariel Prestia^232^, Pablo Galeano^232^, Eduardo M. Castaño^232^, Sandra Germani^233^, Carlos Reyes Toso^233^, Matias Rojo^233^, Carlos Ingino^233^, Carlos Mangone^233^, Sebastiaan Engelborghs^234,235,236,237^, Tagliavini Fabrizio^238^, Sune Fallgaard Nielsen^239^, Lucia Farotti^240^, Chiara Fenoglio^241^, Geert Jan Biessels^242^, Seth Love^243^, Patrick G. Kehoe^243^, Florence Pasquier^244^, Christine Van Broeckhoven^2,3,245^, David C. Rubinsztein^246^, Stefan Teipel^247^, Nathalie Fievet^1^, Vincent Deramecourt^244^, Charlotte Forsell^102,118^, Håkan Thonberg^102,118^, Maria Bjerke^69^, Ellen De Roeck^69^, María Teresa Martínez-Larrad^2248^, Natividad Olivar^233^, Mohsen Ghanbari^11^, Perminder Sachdev^29^, Karen Mather^29^, Frank Jessen^8,16^, M. Arfan Ikram^11^, Alexandre de Mendonça^89^, Jakub Hort^128,129^, Tsolaki Magda^30,31^, Philippe Amouyel^1^, Julie Williams^13^, Ruth Frikke-Schmidt^144,156^, Jordi Clarimon^10,24^, Jean-François Deleuze^14^, Giacomina Rossi^85^, Ole A. Andreassen^40^, Martin Ingelsson^20^, Mikko Hiltunen^17^, Kristel Sleegers^2,3,4^, Cornelia M. van Duijn^11,12^, Rebecca Sims^13^, Wiesje M. van der Flier^5^, Agustín Ruiz^9,10^, Alfredo Ramirez^7,8,16,249^, Jean-Charles Lambert^1^

1. Univ. Lille, Inserm, CHU Lille, Institut Pasteur de Lille, U1167-RID-AGE facteurs de risque et déterminants moléculaires des maladies liés au vieillissement, Lille, France
2. Complex Genetics of Alzheimer's Disease Group, VIB Center for Molecular Neurology, VIB, Antwerp, Belgium
3. Laboratory of Neurogenetics, Institute Born - Bunge, Antwerp, Belgium
4. Department of Biomedical Sciences, University of Antwerp, Neurodegenerative Brain Diseases Group,
5. Alzheimer Center Amsterdam, Department of Neurology, Amsterdam Neuroscience, Vrije Universiteit Amsterdam, Amsterdam UMC, Amsterdam, The Netherlands
6. Department of Complex Trait Genetics, Center for Neurogenomics and Cognitive Research, Amsterdam, The Netherlands
7. Division of Neurogenetics and Molecular Psychiatry, Department of Psychiatry and Psychotherapy, University of Cologne, Medical Faculty, Cologne, Germany.
8. Department of Neurodegenerative Diseases and Geriatric Psychiatry, University Hospital Bonn, Bonn, Germany
9. Research Center and Memory clinic Fundació ACE, Institut Català de Neurociències Aplicades, Universitat Internacional de Catalunya, Barcelona, Spain
10. CIBERNED, Network Center for Biomedical Research in Neurodegenerative Diseases, National Institute of Health Carlos III, Madrid, Spain
11. Department of Epidemiology, ErasmusMC, Totterdam, The Netherlands
12. Nuffield Department of Population Health Oxford University, Oxford, UK
13. MRC Centre for Neuropsychiatric Genetics and Genomics, , Division of Psychological Medicine and Clinical
14. Université Paris-Saclay, CEA, Centre National de Recherche en Génomique Humaine, 91057, Evry, France
15. German Center for Neurodegenerative Diseases (DZNE Bonn), Bonn, Germany
16. Section Genomics of Neurdegenerative Diseases and Aging, Department of Human Genetics Amsterdam, The Netherlands
17. Institute of Biomedicine, University of Eastern Finland, Kuopio, Finland
18. Brain Institute, Federal University of Rio Grande do Norte, Av. Nascimento de Castro 2155 Natal, Brazil
19. LACDR, Leiden, The Netherlands
20. Dept.of Public Health and Carins Sciences / Geriatrics, Uppsala University, Sweden
21. Centre of Age-Related Medicine, Stavanger University Hospital, Norway
22. Institute of Psychiatry, Psychology & Neuroscience, PO 70, 16 De Crespigny Park, London, UK
23. Department of Surgery, Biochemistry and Molecular Biology, School of Medicine, University of Málaga, Málaga, Spain.
24. Department of Neurology, II B Sant Pau, Hospital de la Santa Creu i Sant Pau, Universitat Autònoma de Barcelona, Barcelona, Spain.
25. Fundació Docència i Recerca MútuaTerrassa and Movement Disorders Unit, Department of Neurology, University Hospital MútuaTerrassa, Terrassa 08221, Barcelona, Spain
26. Memory Disorders Unit, Department of Neurology, Hospital Universitari Mutua de Terrassa, Terrassa, Barcelona, Spain.
27. Laboratorio de Genética. Hospital Universitario Central de Asturias, Oviedo, Spain
28. Servicio de Neurología HOspital Universitario Central de Asturias- Oviedo  and Instituto de Investigación Biosanitaria del Principado de Asturias, Oviedo, Spain
29. Centre for Healthy Brain Ageing, School of Psychiatry, Faculty of Medicine, University of New South Wales, Sydney, Australia
30. 1st Department of Neurology, Medical school, Aristotle University of Thessaloniki, Thessaloniki, Makedonia, Greece
31. Alzheimer Hellas, Thessaloniki, Makedonia, Greece
32. School of Medicine and Surgery, University of Milano-Bicocca, Italy
33. Neurology Unit, "San Gerardo" hospital, Monza, Italy
34. Fondazione IRCCS Ca' Granda, Ospedale Policlinico, Milan, Italy
35. Department of Laboratory Diagnostics, III Laboratory of Analysis, Brescia Hospital, Brescia, Italy
36. Unitat Trastorns Cognitius, Hospital Universitari Santa Maria de Lleida, Lleida, Spain
37. Institut de Recerca Biomedica de Lleida (IRBLLeida), Lleida, Spain
38. Department of Clinical Sciences and Community Health, University of Milan, Italy
39. Geriatic Unit, Fondazione Cà Granda, IRCCS Ospedale Maggiore Policlinico, Milan, Italy
40. NORMENT Centre, University of Oslo, Oslo, Norway
41. Université de Paris, EA 4468, APHP, Hôpital Broca, Paris, France
42. Laboratory of Neuropsychiatry, Department of Clinical and Behavioral Neurology, IRCCS Santa Lucia Foundation, Rome, Italy
43. Servei de Neurologia, Hospital Universitari i Politècnic La Fe, Valencia, Spain.
44. CIEN Foundation/Queen Sofia Foundation Alzheimer Center, Madrid, Spain
45. Molecular Markers Laboratory, IRCCS Istituto Centro San Giovanni di Dio Fatebenefratelli, Brescia, Italy
46. Univ. Montpellier, Inserm U1061, Neuropsychiatry: epidemiological and clinical research, PSNREC, Montpellier, France
47. Department of Neuroscience, Psychology, Drug Research and Child Health University of Florence, Florence Italy
48. Azienda Ospedaliero-Universitaria Careggi, Florence, Italy
49. MAC - Memory Clinic, IRCCS Istituto Centro San Giovanni di Dio Fatebenefratelli, Brescia
50. Geriatrics Unit Fondazione Policlinico A. Gemelli IRCCS, Rome , Italy
51. Centre for Neurodegenerative Disorders, Department of Clinical and Experimental Sciences, University of Brescia, Brescia, Italy
52. Department of Neuroscience “Rita Levi Montalcini”, University of Torino, Torino, Italy
53. Experimental Neuro-psychobiology Laboratory,Department of Clinical and Behavioral Neurology, IRCCS Santa Lucia Foundation, Rome, Italy
54. Department of Neurology and Clinical Neurophysiology, University Hospital of Trondheim, Trondheim, Norway
55. Department of Neuromedicine and Movement Science, Norwegian University of Science and Technology, Trondheim, Norway
56. Dementia Centre for Research Collaboration, School of Psychiatry, University of New South Wales, Sydney, Australia
57. Biosciences, School of Science and Technology, Nottingham Trent University, Nottingham UK
58. Centro de Neuropsiquiatría y Neurología de la Conducta (CENECON), Facultad de Medicina, Universidad de Buenos Aires (UBA), C.A.B.A, Buenos Aires, Argentina.
59. Departamento Ciencias Fisiológicas UAII, Facultad de Medicina, UBA, C.A.B.A, Buenos Aires, Argentina.
60. Hospital Interzonal General de Agudos Eva Perón, San Martín, Buenos Aires, Argentina.
61. Unidad de Trastornos del Movimiento, Servicio de Neurología y Neurofisiología. Instituto de Biomedicina de Sevilla (IBiS), Hospital Universitario Virgen del Rocío/CSIC/Universidad de Sevilla, Seville, Spain
62. Institute for Stroke and Dementia Research, Klinikum der Universität München, Ludwig-Maximilians-Universität LMU, Munich, Germany.
63. German Center for Neurodegenerative Diseases (DZNE, Munich), Munich, Germany.
64. Faculty of Medical & Health Sciences, University of Auckland, New Zealand
65. Wales Centre for Ageing & Dementia Research, Swansea University, Wales, New Zealand
66. UFIEC, Instituto de Salud Carlos III, , Madrid, Spain
67. Inserm, Bordeaux Population Health Research Center, UMR 1219, Univ. Bordeaux, ISPED, CIC 1401-EC, Univ Bordeaux,Bordeaux, France
68. CHU de Bordeaux, Pole santé publique, Bordeaux, France
69. Grupo de Medicina Xenómica, Centro Nacional de Genotipado (CEGEN-PRB3-ISCIII). Universidade de Santiago de Compostela, Santiago de Compostela, Spain.
70. Fundación Pública Galega de Medicina Xenómica- CIBERER-IDIS, University of Santiago de Compostela, Santiago de Compostela, Spain.
71. Institute of Gerontology and Geriatrics, Department of Medicine and Surgery, University of Perugia Perugia, Italy
72. Normandie Univ, UNIROUEN, Inserm U1245 and CHU Rouen, Department of Genetics and CNR-MAJ, Rouen, France
73. Unit of Clinical Pharmacology, University Hospital of Cagliari, Cagliari, Italy
74. Radboudumc Alzheimer Center, Department of Geriatrics, Radboud University Medical Center, Nijmegen, the Netherlands
75. Institute for Regenerative Medicine, University of Zürich, Schlieren, Switzerland
76. Unidad Clínica de Enfermedades Infecciosas y Microbiología. Hospital Universitario de Valme, Sevilla, Spain
77. Department of Neuroscience, Catholic University of Sacred Heart, Fondazione Policlinico Universitario A. Gemelli IRCCS, Rome, Italy
78. University of Bari, “A. Moro”, Bari, Italy
79. School of Medicine, University of Thessaly, Larissa, Greece
80. University Bordeaux, Inserm, Bordeaux Population Health Research Center, France
81. Department of Neurology, University Medical Center Groningen, the Netherlands
82. Department of Neurology, Bordeaux University Hospital, Bordeaux, France
83. Department of Psychiatry, Psychosomatics and Psychotherapy, Center of Mental Health, University Hospital, Wuerzburg
84. UKDRI@ Cardiff, School of Medicine, Cardiff University, Cardiff, UK
85. Munich Cluster for Systems Neurology (SyNergy), Munich, Germany.
86. Technical University of Munich, School of Medicine, Klinikum rechts der Isar, Department of Psychiatry and Psychotherapy, Munich, Germany
87. Institute of Cognitive Neurology and Dementia Research (IKND), Otto-Von-Guericke University, Magdeburg, Germany.
88. German Center for Neurodegenerative Diseases (DZNE), Magdeburg, Germany.
89. Faculty of Medicine, University of Lisbon, Portugal
90. Department of Psychiatry, Social Medicine Center East- Donauspital, Vienna, Austria
91. Institute of Clinical Medicine, University of Oslo, Oslo, Norway.
92. Dementia Research Centre, UCL Queen Square Institute of Neurology, London, United Kingdom
93. Unidad de Demencias, Servicio de Neurología y Neurofisiología. Instituto de Biomedicina de Sevilla (IBiS), Hospital Universitario Virgen del Rocío/CSIC/Universidad de Sevilla, Seville, Spain
94. Instituto de Investigacion Sanitaria ‘Hospital la Paz’ (IdIPaz), Madrid, Spain
95. Centro de Biología Molecular Severo Ochoa (UAM-CSIC), Madrid, Spain
96. Hospital Universitario la Paz, Madrid, Spain
97. Department of geriatric Psychiatry, Central Institute for Mental Health, Mannheim, University of Heidelberg, Germany
98. Alzheimer Research Center & Memory Clinic, Andalusian Institute for Neuroscience, Málaga, Spain.
99. Hospital Universitario Ramon y Cajal, IRYCIS, Madrid
100. Department of Psychiatry and Psychotherapy, Medical University of Vienna, Vienna, Austria
101. CAEBI, Centro Andaluz de Estudios Bioinformáticos, Sevilla, Spain.
102. Karolinska Institutet, Center for Alzheimer Research, Department NVS, Division of Neurogeriatrics, Stockholm, Sweden
103. Aging Research Center, Department of Neurobiology, Care Sciences and Society, Karolinska Institutet and Stockholm University, Stockholm, Sweden
104. Institute of Public Health, University of Cambridge, UK
105. Department of Child and Adolescent Psychiatry and Psychotherapy, University Hospital of Psychiatry Zurich, University of Zurich, Zurich, Switzerland
106. Neuroscience Center Zurich, University of Zurich and ETH Zurich, Switzerland
107. Zurich Center for Integrative Human Physiology, University of Zurich, Switzerland
108. Human Genetics, School of Life Sciences, Life Sciences Building, University Park, University of Nottingham, Nottingham, UK
109. A.I Virtanen Institute for Molecular Sciences, University of Eastern Finland, Kuopio, Finland
110. Department of Neurology, Medical School, University of Cyprus, Cyprus
111. Sorbonne University, GRC n° 21, Alzheimer Precision Medicine Initiative (APMI), AP-HP, Pitié-Salpêtrière Hospital, Boulevard de l'hôpital, Paris, France
112. Eisai Inc., Neurology Business Group, 100 Tice Blvd, Woodcliff Lake, NJ 07677, USA
113. Reta Lila Weston Research Laboratories, Department of Molecular Neuroscience, UCL Institute of Neurology, London, UK.
114. Institute of Human Genetics, University of Bonn, School of Medicine & University Hospital Bonn, Bonn, Germany
115. Insitute of Clinical Medicine - Neurology, University of Eastern, Kuopio, Finland
116. Institute of Clinical Medicine – Internal Medicine, University of Eastern Finland, Kuopio, Finland
117. Clinical and Experimental Science, Faculty of Medicine, University of Southampton, Southampton, UK.
118. Unit for Hereditary dementias, Karolinska University Hospital-Solna, Stockholm, Sweden
119. Division of Clinical Geriatrics, Center for Alzheimer Research, Care Sciences and Society (NVS), Karolinska Institutet, Stockholm, Sweden
120. Institute of Public Health and Clinical Nutrition, University of Eastern Finland, Kuopio, Finland
121. Neuroepidemiology and Ageing Research Unit, School of Public Health, Imperial College London, London, United Kingdom
122. Stockholms Sjukhem, Research & Development Unit, Stockholm, Sweden
123. Department of Psychiatry and Psychotherapy, Universitätsklinikum Erlangen, and Friedrich-Alexander Universität Erlangen-Nürnberg, Erlangen, Germany.
124. Laboratory of Cognitive Neuroscience, School of Psychology, Aristotle University of Thessaloniki, Thessaloniki, Greece
125. Neurology Service, Marqués de Valdecilla University Hospital (University of Cantabria and IDIVAL), Santander, Spain.
126. Stockholm Gerontology Research Center, Stockholm, Sweden
127. Public Health Promotion Unit, Finnish Institute for Health and Welfare, Helsinki, Finland
128. Memory Clinic, Department of Neurology, Charles University, 2nd Faculty of Medicine and Motol University Hospital, Czech Republic
129. International Clinical Research Center, St. Anne’s University Hospital Brno, Brno, Czech Republic
130. Department of Neurology. Hospital Universitario Donostia. OSAKIDETZA-Servicio Vasco de Salud, San Sebastian, Spain
131. School of Health Sciences, Bangor University, UK
132. Unit of Neurology, University of Parma and AOU, Parma, Italy
133. Institute of Neurology, Catholic University of the Sacred Heart , Rome, Itlay
134. MRC Prion Unit at UCL, UCL Institute of Prion Diseases, London, UK
135. Clinic of Neurology, UH "Alexandrovska", Medical University - Sofia, Sofia, Bulgaria
136. Institute for Urban Public Health, University Hospital of University Duisburg-Essen, Essen, Germany
137. Department of Neurology, ErasmusMC, Rotterdam, The Netherlands
138. Neurological Tissue Bank of the Biobanc-Hospital Clinic-IDIBAPS, Institut d'Investigacions Biomèdiques August Pi i Sunyer, Barcelona, Spain.
139. Alzheimer’s disease and other cognitive disorders Unit. Neurology Department, Hospital Clinic , Barcelona, Spain
140. Laboratory of Brain Aging and Neurodegeneration- FIL-CONICET, Buenos Aires, Argentina
141. Human Genetics, School of Life Sciences, University of Nottingham, UK
142. IRCCS Fondazione Don Carlo Gnocchi, Florence, Italy
143. Department of Clinical Biochemistry, Herlev and Gentofte Hospital, Herlev, Denmark
144. Department of Clinical Medicine, University of Copenhagen, Copenhagen, Denmark
145. DIMEC, University of Parma, Parma, Italy
146. Institut de Biomedicina de València-CSIC (valència, Spain) CIBERNED.
147. Unitat Mixta de de Neurología y Genética, Institut d'Investigació Sanitària La Fe (València, Spain)
148. Univ. Lille, CNRS, Inserm, CHU Lille, Institut Pasteur de Lille, US 41-UMS 2014-PLBS, bilille, Lille, France.
149. Institute of Psychiatry and Psychotherapy, Charité-Universitätsmedizin Berlin, Corporate Member of Freie Universität Berlin, Humboldt-Universität Zu Berlin, and Berlin Institute of Health, Berlin, Germany.
150. German Center for Neurodegenerative Diseases (DZNE), Berlin, Germany.
151. Department of Biomedical Sciences, University of Cagliari, Italy
152. CHUV, Old Age Psychiatry, Department of Psychiatry, Lausanne, Switzerland
153. Old Age Psychiatry, Department of Psychiatry, Lausanne University Hospital, Lausanne, Switzerland
154. Department of Geriatric Psychiatry, University Hospital of Psychiatry Zürich, Zürich, Switzerland
155. Department of Neuropsychiatry and Laboratory of Molecular Psychiatry, Charité, Charitéplatz 1, 10117 Berlin, Germany
156. Department of Clinical Biochemistry, Rigshospitalet, Copenhagen, Denmark
157. Maastricht University, Department of Psychiatry & Neuropsychologie, Alzheimer Center Limburg, Maastricht, the Netherlands
158. Depatamento de Especialidades Quirúrgicas, Bioquímica e Inmunología. Facultad de Medicina. Universidad de Málaga. Málaga, Spain
159. Delft Bioinformatics Lab, Delft University of Technology, Delft, The Netherlands
160. Institute of Social Medicine, Occupational Health and Public Health, University of Leipzig, 04103 Leipzig, Germany.
161. Center of Mental Health, Clinic and Policlinic of Psychiatry, Psychosomatics and Psychotherapy, University Hospital of Würzburg, Wuerzburg, Germany
162. Department of Research and Innovation, Helse Fonna, Haugesund Hospital, Haugesund, Norway.
163. The University of Bergen, Institute of Clinical Medicine (K1), Bergen Norway
164. Departamento de Especialidades Quirúrgicas, Bioquímicas e Inmunología, School of Medicine, University of Málaga, Málaga, Spain.
165. Department of Neuroscience and Mental Health, AOU Città della Salute e della Scienza di Torino, Torino, Italy
166. Athens Association of Alzheimer’s disease and Related Disorders, Athens, Greece
167. Department of Geriatrics, St. Olav’s Hospital, Trondheim University Hospital, Norway
168. Department of Immunology, Hospital Universitario Doctor Negrín, Las Palmas de Gran Canaria, Spain.
169. Neurology department-Hospital Clínic, IDIBAPS, Universitat de Barcelona, Barcelona, Spain.
170. Taub Institute for Research in Alzheimer’s Disease and the Aging Brain, The Gertrude H. Sergievsky Center, Depatment of Neurology, Columbia University, New York, NY
171. 1st Department of Neurology, Aiginition Hospital, National and Kapodistrian University of Athens, Medical School, Greece
172. LVR-Hospital Essen, Department of Psychiatry and Psychotherapy, Medical Faculty, University of Duisburg-Essen, Virchowstr. 174, 45147 Essen, Germany
173. Department of Primary Medical Care, University Medical Centre Hamburg-Eppendorf, 20246 Hamburg, Germany.
174. Institute of Medical Biometry, Informatics and Epidemiology, University Hospital of Bonn, Bonn, Germany.
175. Department of Geriatric Medicine, Oslo University Hospital, Oslo, Norway
176. Laboratory for Advanced Hematological Diagnostics, Department of Hematology and Stem Cell Transplant, Lecce, Italy
177. Department of Biomedical Sciences, Section of Neuroscience and Clinical Pharmacology, University of Cagliari, Italy
178. Department of Psychiatry, Namsos Hospital, Namsos, Norway
179. Department of Internal medicine and Biostatistics, ErasmusMC, Rooterdam, The Netherlands
180. Department of Radiology&Nuclear medicine, ErasmusMC, Totterdam, The Netherlands
181. Laboratory for Cognitive Neurology, Department of Neurosciences, University of Leuven, Belgium
182. Neurology Department, University Hospitals Leuven, Leuven, Belgium
183. Department of Psychiatry and Psychotherapy, University Medical Center Goettingen, Goettingen, Germany
184. Department of Psychiatry, Harvard Medical School, McLean Hospital, Belmont, MA, USA
185. Normandie Univ, UNIROUEN, Inserm U1245, CHU Rouen, Department of Neurology and CNR-MAJ, F 76000, Normandy Center for Genomic and Personalized Medicine, Rouen, France
186. German Center for Neurodegenerative Diseases (DZNE), Goettingen, Germany
187. Medical Science Department, iBiMED, Aveiro, Portugal
188. Department of Nutrition and Diatetics, Harokopio University, Athens, Greece
189. Neurosciences Area. Instituto Biodonostia. San Sebastian, Spain
190. Department of Clinical Biochemistry, Hematology and Immunology, Na Homolce Hospital, Prague, Czech republic
191. Institute of Gerontology and Geriatrics, Department of Medicine, University of Perugia Perugia (Italy)
192. Insitute of Biomedicine, University of Eastern Finland, Finland
193. Center for Life Course Health Research, University of Oulu, Oulu, Finland
194. Medical Research Center Oulu, Oulu University Hospital, Oulu, Finland
195. University of Helsinki and Helsinki University Hospital, Helsinki, Finland
196. Division of Psychological Medicine and Clinial Neurosciences, MRC Centre for Neuropsychiatric Genetics and Genomics, Cardiff University, UK
197. Kings College London, Institute of Psychiatry, Psychology and Neuroscience, UK
198. Division of Psychiatry, University College London, UK
199. Institute of Public Health, University of Cambridge, Cambridge, UK
200. Institute of Genetics, Queens Medical Centre, University of Nottingham, Nottingham, UK
201. XXX
202. Ageing Group, Centre for Public Health, School of Medicine, Dentistry and Biomedical Sciences, Queen's University Belfast, UK
203. The Wellcome Trust Sanger Institute, Wellcome Trust Genome Campus, Hinxton, Cambridge, UK.
204. Dementia Research Centre, Department of Neurodegenerative Disease, UCL Institute of Neurology, London, UK
205. Mercer's Institute for Research on Ageing, St James' Hospital, Dublin, Ireland
206. Department of Molecular Neuroscience, UCL, Institute of Neurology, London, UK
207. School of Biotechnology, Dublin City University, Dublin, Ireland
208. Centre for Public Health, School of Medicine, Dentistry and Biomedical Sciences, Queens University, Belfast, UK
209. Department of Psychiatry, University of Oxford, Oxford, UK
210. Department of Basic and Clinical Neuroscience, Institute of Psychiatry, Psychology and Neuroscience, Kings College London, London UK
211. Genetic Epidemiology, QIMR Berghofer Medical Research Institute, Herston, Queensland, Australia
212. Division of Neuroscience and Experimental Psychology, School of Biological Sciences, Faculty of Biology, Medicine and Health, University of Manchester, Manchester Academic Health Science Centre, Manchester M13 9PT, UK
213. Oxford Project to Investigate Memory and Ageing (OPTIMA), University of Oxford, Level 4, John Radcliffe Hospital, Oxford, UK
214. Department of Mental Health Sciences, University College London, London, UK
215. Ageing Group, Centre for Public Health, School of Medicine, Dentistry and Biomedical Sciences, Queen’s University Belfast, Belfast, UK.
216. Dementia Research Centre, UCL, London, UK
217. Servei de Neurologia. Hospital Clínic Universitari de València, Spain
218. Department of Radiology, University Hospital Bonn, Bonn, Germany
219. German Center for Neurodegenerative Diseases (DZNE), Tübingen, Germany
220. Section for Dementia Research, Hertie Institute for Clinical Brain Research and Department of Psychiatry, Tübingen, Germany
221. Department of Psychiatry and Psychotherapy, University Hospital, LMU Munich, Munich, Germany
222. Service of Neurology, University Hospital Marqués de Valdecilla, IDIVAL, University of Cantabria, Santander, Spain
223. Molecular Medicine Center, Department of Medical chemistry and biochemistry, Medical University of Sofia, Bulgaria
224. Department of Psychiatry, Psychosomatics and Psychotherapy, Center of Mental Health, University Hospital of Würzburg, Germany
225. ENYS (Estudio en Neurociencias y Sistemas Complejos) CONICET- Hospital El Cruce "Nestor Kirchner"- UNAJ, Argentina
226. HIGA Eva Perón, Buenes Aires, Agentina
227. Neurología Clinica, Buenes Aires, Agentina
228. Dirección de Atención de Adultos Mayores del Min. Salud Desarrollo Social y Deportes de la Pcia. de Mendoza, Argentina
229. Laboratorio de Genética Forense del Ministerio Público de la Pcia. de La Pampa, Argentina
230. Fundacion Sinapsis, Santa Rosa, Argentina
231. Hospital Dr. Lucio Molas, Santa Rosa; Fundacion Ayuda Enfermo Renal y Alta Complejidad (FERNAC), Santa Rosa, Argentina
232. Laboratory of Brain Aging and Neurodegeneration- FIL, Buneos Aires, Argentina
233. Centro de Neuropsiquiatría y Neurología de la Conducta (CENECON), Facultad de Medicina, Universidad de Buenos Aires (UBA), C.A.B.A, Buenos Aires, Argentina
234. Center for Neurosciences, Vrije Universiteit Brussel (VUB), Brussels, Belgium
235. Reference Center for Biological Markers of Dementia (BIODEM), Institute Born-Bunge, University of Antwerp, Antwerp, Belgium
236. Institute Born-Bunge, University of Antwerp, Antwerp, Belgium
237. Department of Neurology, UZ Brussel, Brussels, Belgium
238. Fondazione IRCCS, Istituto Neurologico Carlo Besta, Milan Italy
239. Department of Clinical Biochemistry, Herlev and Gentofte Hospital, Herlev Denmark
240. Centre for Memory Disturbances, Lab of Clinical Neurochemistry, Section of Neurology, University of Perugia, Italy
241. University of Milan, Milan, Italy
242. Department of Neurology, UMC Utrecht Brain Center, Utrecht, the Netherlands
243. Translational Health Sciences, Bristol Medical School, University of Bristol, Bristol, BS16 1LE, UK
244. Univ Lille Inserm 1172, CHU Clinical and Research Memory Research Centre (CMRR) of Distalz, Licend, Lille France
245. Neurodegenerative Brain Diseases Group, VIB Center for Molecular Neurology, VIB, Antwerp, Belgium
246. Cambridge Institute for Medical Research and UK Dementia Research Institute, University of Cambridge, Cambridge, UK
247. German Center for Neurodegenerative Diseases (DZNE), Rostock, Germany
248. Centro de Investigación Biomédica en Red de Diabetes y Enfermedades Metabólicas Asociadas, CIBERDEM, Spain, Hospital Clínico San Carlos, Madrid, Spain
249. Glenn Biggs Institute for Alzheimer’s and Neurodegenerative Diseases, San Antonio, TX, USA

**The GR@ACE study group**

Aguilera N^1^, Alarcon E^1^, Alegret M^1,2^, Boada M^1,2^, Buendia M^1^, Cano A^1^, Cañabate P^1,2^, Carracedo A^4,5^, Corbat´on-Anchuelo A^6^, de Rojas I^1^, Diego S^1^, Espinosa A^1,2^, Gailhajenet A^1^, García-González P^1,2^, Guitart M^1^, Gonz´alez-P´erez A^7^, Ibarria M^1^, Lafuente A^1^, Macias J^8^, Maro~nas O^4^, Mart´ın E^1^, Mart´ınez MT^6^, Marqui´e M^1,2^, Montrreal L^1^, Moreno- Grau S^1,2^, Moreno M^1^, R. Nuñez-Llaves R^1^, Olivé C^1^ , Orellana A^1^, Ortega G^1,2^, Pancho A^1^, Pelej`a E^1^, P´erez-Cordon A^1^, Pineda JA^8^, Puerta R^1^, Preckler S^1^, Quintela I^3^, Real LM^3,8^, Rosende- Roca M^1^, Ruiz A^1,2^, S´aez ME^7^, Sanabria A^1,2^, Serrano-Rios M^6^, Sotolongo-Grau O^1^, T´arraga L^1,2^, Valero S^1,2^, Vargas L^1^

1 Research Center and Memory clinic. ACE Alzheimer Center Barcelona, Universitat Internacional de Catalunya, Spain.

2. CIBERNED, Center for Networked Biomedical Research on Neurodegenerative Diseases, National Institute of Health Carlos III, Ministry of Economy and Competitiveness, Spain,

^3.^ Dep. of Surgery, Biochemistry and Molecular Biology, School of Medicine. University of M´alaga. M´alaga, Spain,

4. Grupo de Medicina Xen´omica, Centro Nacional de Genotipado (CEGEN-PRB3-ISCIII). Universidad de Santiago de Compostela, Santiago de Compostela, Spain.

5. Fundaci´on P´ublica Galega de Medicina Xen´omica- CIBERER-IDIS, Santiago de Compostela, Spain.

6. Centro de Investigaci´on Biom´edica en Red de Diabetes y Enfermedades Metab´olicas Asociadas, CIBERDEM, Spain, Hospital Clínico San Carlos, Madrid, Spain,

7. CAEBI. Centro Andaluz de Estudios Bioinform´aticos, Sevilla, Spain

8. Unidad Clínica de Enfermedades Infecciosas y Microbiología. Hospital Universitario de Valme, Sevilla, Spain.

**DEGESCO consortium**

Adarmes-G´omez AD^1,2^, Alarc´on-Martín E^3^, Alonso MD^4^, Álvarez I^5^, Álvarez V^6,7^, Amer-Ferrer G^8^, Antequera M^9^, Ant´unez C^9^, Baquero M^10^, Bernal M^11^, Blesa R^2,12^, Boada M^2,3^, Buiza-Rueda D^1,2^, Bullido MJ^2,14,15^, Burguera JA^10^, Calero M^2,16,17^, Carrillo F^1,2^, Carri´on-Claro M^1,2^, Casajeros MJ^18^, Clarim´on J^2,12^, Cruz-Gamero JM^13^, de Pancorbo MM^19^, de Rojas I^2,3^, del Ser T^15^, Diez-Fairen M^5^, Escuela R^1,2^, Garrote-Espina L^1,2^, Fortea J^2,12^, Franco E^11^, Frank-Garc´ıa A^2,15,20^, García-Alberca JM^21^, Garcia Madrona S^17^, Garcia-Ribas G^17^, G´omez-Garre P^1,2^, Hevilla S^21^, Jes´us S^1,2^, Labrador Espinosa MA^1,2^, Lage C^2,22^, Legaz A^9^, Lle´o A^2,12^, L´opez de Mun´ain A^23^, L´opez-Garc´ıa S^2,22^, Macias-García D^1,2^, Manzanares S^8,24^, Mar´ın M^11^, Mar´ın-Muñoz J^9^, Mar´ın T^21^, Marqui´e M^2,3^, Mart´ın Montes A^2,14,20^, Mart´ınez B^9^, Mart´ınez C^7,25^, Mart´ınez V^9^, Mart´ınez-Lage A´lvarez P^26^, Medina M^2,15^, Mendioroz Iriarte M^27^, Men´endez- Gonz´alez M^7,28^, Mir P^1,2^, Montrreal L^3^, Orellana A^3^, Pastor P^5^, P´erez Tur J^2,29,30^, Periñán-Tocino T^1,2^, Pineda-Sánchez R^1,2^, Piñol Ripoll G^2,31^, R´abano A^2,16,32^, Real de As´ua D^33^, Rodrigo S^11^, Rodr´ıguez-Rodr´ıguez E^2,22^, Royo JL^13^, Ruiz A^2,3^, Sanchez del Valle D´ıaz R^34^, S´anchez-Juan P^16^, Sastre I^2,14^, Sotolongo-Grau O^3^, Valero S^2,3^, Vicente MP^9^, Vigo-Ortega R^1,2^, Vivancos L^9^

1. Unidad de Trastornos del Movimiento, Servicio de Neurolog´ıa y Neurofisiolog´ıa. Instituto de Biomedicina de Sevilla (IBiS), Hospital Universitario Virgen del Rocío/CSIC/Universidad de Sevilla, Seville, Spain,
2. CIBERNED, Network Center for Biomedical Research in Neurodegenerative Diseases, National Institute of Health Carlos III, Spain,
3. Research Center and Memory clinic. ACE Alzheimer Center Barcelona, Universitat Internacional de Catalunya, Spain,
4. Servei de Neurologia. Hospital Clínic Universitari de València, Spain.
5. Fundaci´o per la Recerca Biom`edica i Social M´utua Terrassa, and Memory Disorders Unit, Department of Neurology, Hospital Universitari Mutua de Terrassa, University of Barcelona School of Medicine, Terrassa, Barcelona, Spain,
6. Laboratorio de Gen´etica Hospital Universitario Central de Asturias, Oviedo, Spain
7. Instituto de Investigaci´on Biosanitaria del Principado de Asturias (ISPA), Oviedo, Spain
8. Department of Neurology, Hospital Universitario Son Espases, Palma, Spain,
9. Unidad de Demencias. Hospital Clínico Universitario Virgen de la Arrixaca, Palma, Spain,
10. Servei de Neurologia, Hospital Universitari i Polit`ecnic La Fe, Velencia, Spain
11. Unidad de Demencias, Servicio de Neurolog´ıa y Neurofisiolog´ıa. Instituto de Biomedicina de Sevilla (IBiS), Hospital Universitario Virgen del Roc´ıo/CSIC/Universidad de Sevilla, Seville, Spain
12. Memory Unit, Neurology Department and Sant Pau Biomedical Research Institute, Hospital de la Santa Creu i Sant Pau, Universitat Aut`onoma de Barce- lona, Barcelona, Spain,
13. Dep. of Surgery, Biochemistry and Molecular Biology, School of Medicine. University of M´alaga. M´alaga, Spain
14. Centro de Biologia Molecular Severo Ochoa (C.S.I.C.-U.A.M.), Universidad Autonoma de Madrid, Madrid, Spain
15. Instituto de Investigacion Sanitaria ‘Hospital la Paz’ (IdIPaz), Madrid, Spain,
16. CIEN Foundation, Queen Sofia Foundation Alzheimer Center, Madrid, Spain
17. Instituto de Salud Carlos III (IS- CIII), Madrid, Spain;
18. ^18^Hospital Universitario Ram´on y Cajal; Madrid, Spain,
19. BIOMICs, País Vasco; Centro de Investigaci´on Lascaray. Universidad del Pa´ıs Vasco UPV/EHU, Vitoria-Gasteiz, Spain
20. Neurology Service, Hospital Universitario La Paz (UAM), Madrid, Spain,
21. Alzheimer Research Center & Memory Clinic. Andalusian Institute for Neuroscience. M´alaga, Spain,
22. Neurology Service, Marqu´es de Valdecilla University Hospital (University of Cantabria and IDIVAL), Santander, Spain,
23. Hospital Donostia de San Sebast´ıan, San Sebast´ıan, Spain
24. Fundaci´on para la Formación e Investigación Sanitarias de la Región de Murcia, Palma Spain
25. Servicio de Neurolog´ıa -Hospital de Cabue~nes-Gij´on, Gijón, Spain
26. Centro de Investigacio´n y Terapias Avanzadas. Fundaci´on CITA-alzheimer, San Sebastian, Spain
27. Navarrabiomed, Pamplona, Spain
28. Servicio de Neurolog´ıa Hospital Universitario Central de Asturias, Oviedo, Spain
29. Unitat de Gen`etica Molecular. Institut de Biomedicina de Val`encia-CSIC, Vencia, Spain
30. Unidad Mixta de Neurologia Gen`etica. Instituto de Investigación Sanitaria La Fe, Valencia, Spain
31. Unitat Trastorns Cognitius, Hospital Universitari Santa Maria de Lleida, Institut de Recerca Biom´edica de Lleida (IRBLLeida), Lleida, Spain
32. BT-CIEN,
33. Hospital Universitario La Princesa, Madrid, Spain,
34. Hospital Cl´ınic Barcelona, Spain

**Demgene**

Alexey A Shadrin^1,2^, Shahram Bahrami^1,2^, Arvid Rongve^3,4^, Geir Bråthen^5,6^, Ingunn Bosnes^7,8^, Eystein Stordal^7,8^, Lavinia Athanasiu^1,2^, Per Selnes^9^, Ingvild Saltvedt^5,10^, Sigrid B. Sando^5,6^, Sverre Bergh^11^, Ingun Ulstein^12^, Srdjan Djurovic^13,14^, Tormod Fladby^9,15^, Dag Aarsland^16,17^, Geir Selbæk^12,15,18^, Ole A. Andreassen^1,2^

**EADI**

Céline Bellenguez^1^, Benjamin Grenier-Boley^1^, Jacques Epelbaum^2^, David Wallon^3^, Didier Hannequin^3^, Florence Pasquier^4^, Claudine Berr^5^, Jean-Francois Dartigues^6^, Dominique campion^7^, Christophe Tzourio^8^, Vincent Dermecourt^4^, Nathalie Fievet^1^, Olivier Hanon^9^, Carole Dufouil^8^, Alexis Brice^10^, Bruno Dubois^11^, Karen Ritchie^5^, Phillippe Amouyel^1^, Jean-Charles Lambert^1^

1. Univ. Lille, Inserm, CHU Lille, Institut Pasteur Lille, U1167-RID-AGE - Facteurs de risque et déterminants moléculaires des maladies liées au vieillissement, F-59000 Lille, France
2. UMR 894, Center for Psychiatry and Neuroscience, INSERM, Université Paris Descartes, F-75000 Paris , France
3. Normandie Univ, UNIROUEN, Inserm U1245, CHU Rouen, Department of Neurology and CNR-MAJ, F 76000, Normandy Center for Genomic and Personalized Medicine, Rouen, France
4. Univ. Lille, Inserm, CHU Lille, UMR1172, Resources and Research Memory Center (MRRC) of Distalz, Licend, Lille France
5. Univ. Montpellier, Inserm U1061, Neuropsychiatry: epidemiological and clinical research, PSNREC, Montpellier, France
6. University Bordeaux, Inserm, Bordeaux Population Health Research Center, France
7. Normandie Univ, UNIROUEN, Inserm U1245 and CHU Rouen, Department of Genetics and CNR-MAJ, Rouen, France
8. University Bordeaux, Inserm, Bordeaux Population Health Research Center, France
9. Université de Paris, EA 4468, APHP, Hôpital Broca, Paris, France
10. Inserm U1127, CNRS UMR7225, Sorbonne Universités, UPMC Univ Paris 06, UMR_S1127, Institut du Cerveau et de la Moelle épinière, F-75013, Paris, France; 22. APHP, Department of genetics, Pitié-Salpêtrière Hospital, 75013, Paris, France
11. Institut de la Mémoire et de la Maladie d'Alzheimer (IM2A), Département de Neurologie, Hôpital de la Pitié-Salpêtrière, AP-HP, Paris, France; Institut des Neurosciences Translationnelles de Paris (IHU-A-ICM), Institut du Cerveau et de la Moelle Epinière (ICM), Paris, France; 26. INSERM, CNRS, UMR-S975, Institut du Cerveau et de la Moelle Epinière (ICM), Paris, France; Sorbonne Universités, Université Pierre et Marie Curie, Hôpital de la Pitié-Salpêtrière, AP-HP, Paris, France

**GERAD**

Denise Harold^1^, Paul Hollingworth^2^, Rebecca Sims^2^, Amy Gerrish^2^, Nicola Denning^2^, Amy Williams^2^, Charlene Thomas^2^, Alun Meggy^2,3^, Rachel Marshall^2^, Chloe Davies^2^, Lauren Luckcuck^2,3^, William Nash^2^, Kimberley Dowzell^2^, Atahualpa Castillo Morales^2,3^, Mateus Bernardo-Harrington^2,3^, Patrick Kehoe^4^, Per Hoffmann^4^, Seth Love^4^, James Turton^5^, Jenny Lord^5^, Kristelle Brown^5^, Kevin Morgan^5^, Emma Vardy^6^, Elizabeth Fisher^7^, Jason D. Warren^7^, Jonathan M. Schott^7^, Martin Rossor^7^, Natalie S. Ryan^7^, Nick C. Fox^7^, Rita Guerreiro^7^, Simon Mead^7^, James Uphill^8^, John Collinge^8^, Michelle Lupton^8^, Ammar Al-Chalabi^9^, Christopher E. Shaw^9^, Nick Bass^10^, Richard Abraham^11^, Reinhard Heun^11^, Heike Kölsch^11^, Britta Schürmann^11^, Frank Jessen^11,17^, Wolfgang Maier^11,17^,André Lacour^12^, Christine Herold^12^, Simon Lovestone^13^, Bernadette McGuinness^14^, David Craig^14^, Janet A. Johnston^14^, Michael Gill^14^, Peter Passmore^14^, Stephen Todd^14^, John Powell^15^, Petra Proitsi^15^, Yogen Patel^15^, Angela Hodges^16^, Tim Becker^17,19^, A. David Smith^20^, Donald Warden^20^, Gordon Wilcock^20^, Robert Clarke^21^, Aoibhinn Lynch^22^, Brian Lawlor^22^, Michael Gill^22, 23^, Andrew McQuillin^24^, Gill Livingston^24^, John Hardy^25^, David C. Rubinsztein^26^, Carol Brayne^27^, Rhian Gwilliam^28^, Panagiotis Deloukas^28^, Yoav Ben-Shlomo^29^, David Mann^30^, Nigel M. Hooper^31^, Stuart Pickering-Brown^31^, Clive Holmes^32^, Rebecca Sussams^32^, Nick Warner^33^, Anthony Bayer^34^, Andrew B. Singleton^35^, Annette M Hartmann^36^, Dan Rujescu^36^, Ina Giegling^36^, Harald Hampel^37, 38^, Martin Dichgans^39^, Isabella Heuser^40^, Dmitriy Drichel^41^, Norman Klopp^42^, Markus M. Nöthen^43, 44^, Manuel Mayhaus^45^, Matthias Riemenschneider^45^, Sabrina Pinchler^45^, Thomas Feulner^45^, Wei Gu^45^, Hendrik van den Bussche^46^, Martin Scherer^46^, Jens Wiltfang^47^, Johannes Kornhuber^48^, Michael Hüll^49^, Lutz Frölich^50^, H-Erich Wichmann^51^, Karl-Heinz Jöckel^52^, Susanne Moebus^52^, Steffi Riedel-Heller^53^, John Kauwe^54^, John Morris^55,58^, Kevin Mayo^55,56,57^, Magda Tsolaki^59^, Michael O’Donovan^2^, Lesley Jones^2^, Michael Owen^2^, Valentina Escott-Price^2^, Alfredo Ramirez^18, 19^, Peter Holmans^2^, Julie Williams^2,3^

1. School of Biotechnology, Dublin City University, Dublin, Ireland.
2. Division of Psychological Medicine and Clinical Neurosciences, Medical Research Council (MRC) Centre for Neuropsychiatric Genetics & Genomics, Cardiff University, Cardiff, UK.
3. UK Dementia Research Institute at Cardiff, Cardiff University, Cardiff, UK.
4. University of Bristol Medical School, Learning & Research level 2, Southmead Hospital, Bristol, UK.
5. Institute of Genetics, Queen’s Medical Centre, University of Nottingham, UK
6. Institute for Ageing and Health, Newcastle University, Biomedical Research Building, Campus for Ageing and Vitality, Newcastle upon Tyne, UK
7. Department of Neurodegenerative Disease, UCL Institute of Neurology, London, UK.
8. Department of Neurodegenerative Disease, MRC Prion Unit at UCL, Institute of Prion Diseases, London, UK
9. MRC Centre for Neurodegeneration Research, Department of Clinical Neuroscience, King’s College London, Institute of Psychiatry, London, UK.
10. Division of Psychiatry, University College London, London, UK.
11. Department of Psychiatry and Psychotherapy, University of Bonn, Bonn, Germany
12. Deutsches Zentrum für Neurodegenerative Erkrankungen (DZNE, Bonn), Bonn, Germany
13. Department of Psychiatry, University of Oxford, Oxford, UK.
14. Ageing Group, Centre for Public Health, School of Medicine, Dentistry and Biomedical Sciences, Queen’s University, Belfast, UK.
15. Department of Basic and Clinical Neuroscience, Institute of Psychiatry, Psychology and Neuroscience, King’s College London, London, UK.
16. Department of Old Age Psychiatry, Institute of Psychiatry, Psychology and Neuroscience, King’s College London, London, UK.
17. German Centre for Neurodegenerative Diseases, Bonn, Germany.
18. Department for Neurodegenerative Diseases and Geriatric Psychiatry, University Hospital Bonn, Bonn, Germany.
19. Institute for Medical Biometry, Informatics and Epidemiology, University of Bonn, Bonn, Germany
20. Oxford Project to Investigate Memory and Ageing (OPTIMA), University of Oxford, Nuffield Department of Clinical Neurosciences, John Radcliffe Hospital, Oxford, UK
21. Oxford Healthy Aging Project, Clinical Trial Service Unit, University of Oxford, Oxford, UK.
22. Mercer’s Institute for Research on Aging, St. James’s Hospital and Trinity College, Dublin, Ireland.
23. St. James’s Hospital and Trinity College, Dublin, Ireland.
24. Department of Mental Health Sciences, University College London, UK.
25. Department of Molecular Neuroscience, UCL, Institute of Neurology, London, UK.
26. Cambridge Institute for Medical Research, University of Cambridge, Cambridge, UK
27. Institute of Public Health, University of Cambridge, Cambridge, UK.
28. The Wellcome Trust Sanger Institute, Hinxton, Cambridge, UK.
29. Population Health Sciences, Bristol Medical School, University of Bristol, Bristol, UK.
30. Clinical Neuroscience Research Group, Greater Manchester Neurosciences Centre, University of Manchester, Salford, UK
31. Division of Neuroscience and Experimental Psychology, School of Biological Sciences, Faculty of Biology, Medicine and Health, University of Manchester, Manchester Academic Health Science Centre, Manchester, UK.
32. Division of Clinical Neurosciences, School of Medicine, University of Southampton, Southampton, UK.
33. Somerset Partnership NHS Trust, Somerset, UK.
34. Institute of Primary Care and Public Health, Cardiff University, University Hospital of Wales, Cardiff, UK.
35. Laboratory of Neurogenetics, National Institute on Aging, National Institutes of Health, Bethesda, MD, 20892, USA.
36. Department of Psychiatry, Martin Luther University Halle-Wittenberg, Halle, Germany.
37. Department of Psychiatry, University of Frankfurt, Frankfurt am Main, Germany.
38. Department of Psychiatry, Ludwig Maximilians University, Munich, Germany.
39. Institute for Stroke and Dementia Research, Klinikum der Universität München, Munich, Germany.
40. Department of Psychiatry and Psychotherapy, Charité University Medicine, Berlin, Germany.
41. Cologne Center for Genomics, University of Cologne, Cologne, Germany.
42. Institute of Epidemiology, Helmholtz Zentrum München, German Research Center for Environmental Health, Neuherberg, Munich, Germany.
43. Institute of Human Genetics, University of Bonn, Bonn, Germany.
44. Department of Genomics, Life & Brain Center, University of Bonn, Bonn, Germany.
45. Department of Psychiatry and Psychotherapy, University Hospital, Saarland, Germany.
46. Institute of Primary Medical Care, University Medical Center Hamburg-Eppendorf, Germany
47. Department of Psychiatry and Psychotherapy, University Medical Center Goettingen, Goettingen, Germany
48. Department of Psychiatry and Psychotherapy, University of Erlangen-Nuremberg, Erlangen, Germany
49. Department of Psychiatry, University of Freiburg, Freiburg, Germany.
50. Central Institute of Mental Health, Medical Faculty Mannheim, University of Heidelberg, Heidelberg, Germany.
51. Institute of Epidemiology, Helmholtz Zentrum München, German Research Center for Environmental Health, Neuherberg, Germany
52. Institute for Medical Informatics, Biometry and Epidemiology, University Hospital of Essen, University Duisburg-Essen, Essen, Germany.
53. Institute of Social Medicine, Occupational Health and Public Health, University of Leipzig, Leipzig, Germany.
54. Departments of Biology, Brigham Young University, Provo, UT, USA.
55. Department of Psychiatry, Washington University School of Medicine, St. Louis, MO, USA.
56. Department of Neurology, Washington University, St. Louis, MO, USA.
57. Department of Genetics, Washington University, St. Louis, MO, USA
58. Hope Center Program on Protein Aggregation and Neurodegeneration, Washington University School of Medicine, St. Louis, MO, USA.
59. Department of Neurology, Medical School, Aristotle University of Thessaloniki, Thessaloniki, Greece.

**Asian Parkinson’s Disease Genetics Consortium (APDGC)**

Jia Nee Foo¹,², Elaine Guo Yan Chew¹,², Sun Ju Chung³, Rong Peng⁴, Yinxia Chao⁵,⁶, Louis CS Tan⁶,⁷, Moses Tandiono¹,², Michelle M Lian¹,², Ebonne Y Ng⁵, Kumar-M. Prakash⁵, Wing-Lok Au⁷, Wee-Yang Meah², Shi Qi Mok², Azlina Ahmad Annuar⁸, Anne YY Chan⁹, Ling Chen¹⁰, Yongping Chen⁴, Beom S Jeon¹¹, Lulu Jiang¹⁰, Jia Lun Lim⁸,¹², Juei-Jueng Lin¹³, Chunfeng Liu¹⁴, Chengjie Mao¹⁴, Vincent Mok⁹, Zhong Pei¹⁰, Hui-Fang Shang⁴, Chang-He Shi¹⁵, Kyuyoung Song¹⁶, Ai Huey Tan¹², Yih-Ru Wu¹⁷, Yu-ming Xu¹⁵, Renshi Xu¹⁸, Yaping Yan¹⁹, Jing Yang¹⁵, Bao Rong Zhang¹⁹, Woon-Puay Koh¹⁰, Shen-Yang Lim¹², Chiea Chuen Khor²,²⁰, Jianjun Liu², Eng-King Tan⁵,⁶

1. Lee Kong Chian School of Medicine, Nanyang Technological University Singapore, 11 Mandalay Road, Singapore 308232, Singapore
2. Human Genetics, Genome Institute of Singapore, A*STAR, 60 Biopolis Street, Singapore 138672, Singapore
3. Department of Neurology, Asan Medical Center, University of Ulsan College of Medicine, Seoul, South Korea
4. Department of Neurology, West China Hospital, Sichuan University, Chengdu, Sichuan Province 610041, Sichuan, People’s Republic of China
5. Department of Neurology, National Neuroscience Institute, Singapore General Hospital, 20 College Road, Singapore 169856, Singapore
6. Duke-National University of Singapore Medical School, 8 College Road, Singapore 169857, Singapore
7. Department of Neurology, National Neuroscience Institute, 11 Jalan Tan Tock Seng, Singapore 308433, Singapore
8. Department of Biomedical Science, Faculty of Medicine, University of Malaya, Kuala Lumpur, Malaysia
9. Margaret K.L. Cheung Research Centre for Management of Parkinsonism, Gerald Choa Neuroscience Centre, Lui Che Woo Institute of Innovative Medicine, Division of Neurology, Department of Medicine and Therapeutics, Prince of Wales Hospital, The Chinese University of Hong Kong, Hong Kong, Hong Kong SAR, People’s Republic of China
10. Department of Neurology, The First Affiliated Hospital,Sun Yat-Sen University, 58 Zhongshan Road II, Guangzhou 510080, People’s Republic of China
11. Department of Neurology, Seoul National University Hospital, 101 Daehak-ro, Jongno-gu, Seoul 110-744, South Korea
12. Department of Medicine and the Mah Pooi Soo and Tan Chin Nam Centre for Parkinson's and Related Disorders, Faculty of Medicine, University of Malaya, Kuala Lumpur, Malaysia
13. Department of Neurology, Chushang Show-Chwan Hospital, No.75 Jishan Road Section 2, Zhushan District, Nantou, Taiwan
14. Department of Neurology, Second Affiliated Hospital of Soochow University, 1055 Sanxiang Road, Suzhou 215004, People’s Republic of China
15. Department of Neurology, The First Affiliated Hospital of Zhengzhou University, Zhengzhou 450000, Henan, People’s Republic of China
16. Department of Biochemistry and Molecular Biology, University of Ulsan College of Medicine, Seoul, South Korea
17. Department of Neurology, Chang Gung Memorial Hospital, Chang Gung University, Taipei 10507, Taiwan
18. Department of Neurology, Jiangxi Provincial People's Hospital, Nanchang 330006, Jiangxi, People’s Republic of China
19. Department of Neurology, Second Affiliated Hospital, College of Medicine, Zhejiang University, Hangzhou 310009, Zhejiang Province, People’s Republic of China
20. Singapore Eye Research Institute, 20 College Road Discovery Tower, Level 6 The Academia, Singapore 169856, Singapore
